# Piercing of the Human Parainfluenza Virus by Nanostructured Surfaces

**DOI:** 10.1021/acsnano.3c07099

**Published:** 2023-12-21

**Authors:** Samson W. L. Mah, Denver P. Linklater, Vassil Tzanov, Phuc H. Le, Chaitali Dekiwadia, Edwin Mayes, Ranya Simons, Daniel J. Eyckens, Graeme Moad, Soichiro Saita, Saulius Joudkazis, David A. Jans, Vladimir A. Baulin, Natalie A. Borg, Elena P. Ivanova

**Affiliations:** † School of Science, STEM College, RMIT University, Melbourne, Victoria 3000, Australia; ‡ CSIRO Manufacturing, Clayton, Victoria 3168, Australia; § Department of Biomedical Engineering, Graeme Clarke Institute, The University of Melbourne, Parkville, Victoria 3010, Australia; ∥ Departament de Química Física i Inorgànica, Universitat Rovira i Virgili, C/Marcel.lí Domingo s/n, Tarragona 43007, Spain; ⊥ RMIT Microscopy and Microanalysis Facility, STEM College,RMIT University, Melbourne, Victoria 3000, Australia; # The KAITEKI Institute Inc., Chiyoda-ku, Tokyo 100-8251, Japan; ○ Optical Science Centre, Swinburne University of Technology, Hawthorn, Melbourne, Victoria 3122, Australia; ★ Nuclear Signalling Laboratory, Department of Biochemistry and Molecular Biology, Monash University, Monash, Victoria 3800, Australia; & School of Health and Biomedical Sciences, RMIT University, Bundoora, Victoria 3083, Australia

**Keywords:** antiviral surfaces, virus−surface interactions, mechanisms of antiviral activity, nanostructured surfaces, biointerfaces, biomimetic surfaces

## Abstract

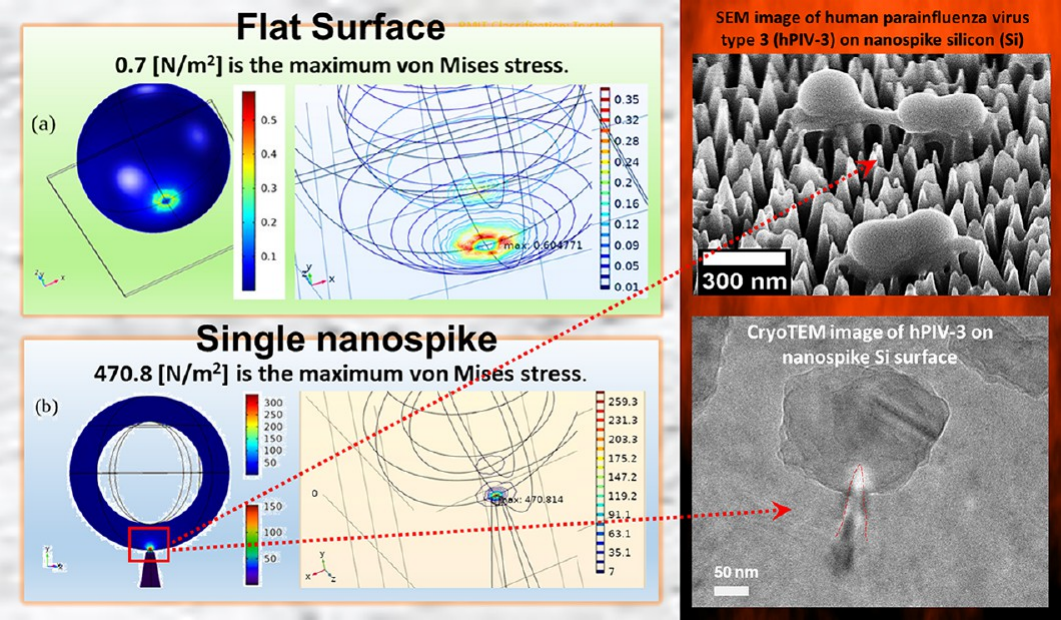

This paper presents a comprehensive experimental and theoretical investigation into the antiviral properties of nanostructured surfaces and explains the underlying virucidal mechanism. We used reactive ion etching to fabricate silicon (Si) surfaces featuring an array of sharp nanospikes with an approximate tip diameter of 2 nm and a height of 290 nm. The nanospike surfaces exhibited a 1.5 log reduction in infectivity of human parainfluenza virus type 3 (hPIV-3) after 6 h, a substantially enhanced efficiency, compared to that of smooth Si. Theoretical modeling of the virus–nanospike interactions determined the virucidal action of the nanostructured substrata to be associated with the ability of the sharp nanofeatures to effectively penetrate the viral envelope, resulting in the loss of viral infectivity. Our research highlights the significance of the potential application of nanostructured surfaces in combating the spread of viruses and bacteria. Notably, our study provides valuable insights into the design and optimization of antiviral surfaces with a particular emphasis on the crucial role played by sharp nanofeatures in maximizing their effectiveness.

## Introduction

The world faces a continual onslaught of viral diseases from both known and emerging pathogens. Human parainfluenza viruses (hPIVs) cause one-third of acute respiratory diseases and are most commonly associated with lower respiratory tract infections in children.^1,2^ Of the four serotypes (hPIV-1–hPIV-4), hPIV-3 is the most virulent, primarily causing infection in immunocompromised individuals and children under 5 years of age.^2−4^ Besides direct transmission (via airborne transmission of expelled aerosols or contact with infected bodily fluids), respiratory viruses, including hPIV-3, are indirectly transmissible via contact with contaminated high-touch traffic surfaces and shared equipment.^5−8^ The transmission of hPIV-3 is believed to be via airborne droplets over short ranges and direct and indirect contact with contaminated surfaces and objects.^9^ No licensed vaccine or antiviral is available for the prevention or treatment of hPIV-3 infections; infections entail a tremendous annual economic loss of $250 million in the United States.^10^

To combat the surface transmission of viruses, chemical design principles and nanotechnology have been utilized to develop composite materials as surface coatings with demonstrated virucidal efficiency. These materials include coatings based on quaternary ammonium compounds (QACs), polysaccharides, and heterocyclic and aromatic polymers.^11−13^ Antiviral polymers must be in direct contact with viral particles to be effective, and constant exposure of the materials to the environment may make them susceptible to erosion and degradation. Additionally, the use of heavy metals and their derivatives, such as gold (Au), silver (Ag), aluminum (Al), copper (Cu), iron oxide (Fe_2_O_3_), zinc oxide (ZnO), magnesium oxide (MgO), and titanium dioxide (TiO_2_), have been intensively studied for their effectiveness against viruses.^14−19^ The viral particles are believed to be inactivated due to metal ion release and the production of reactive oxygen species that can induce damage to membranes and proteins.^20−22^ Unfortunately, various factors impede the widespread application of these technologies to shared surfaces, including concerns associated with their toxicity toward the aquatic environment and human cells.^22,23^ Composite materials containing metal and metal-oxide nanoparticles are also costly to develop, with some requiring UV illumination to be effective and prone to corrosion and disintegration.^24,25^ Thus, there is a need to develop an alternative strategy to combat the surface transmission of viruses. A recent approach is the development of nanostructured antiviral surfaces.^11,26^

It was previously shown that sophisticated nanotextures found in nature can achieve biocidal activity via mechano-responsive mechanisms.^27−30^ Insects such as cicadas, damselflies, and dragonflies, possess a wing surface topography of nanoprotrusions capable of rupturing bacteria and fungi.^27,28,31−33^ The bactericidal efficiency of these nanoprotrusions is influenced by various geometric parameters, such as surface density, height, and width, as well as mechanical properties, including elasticity.^30,34−36^ Studies have demonstrated that most nanoprotrusions exhibit biocompatibility, promoting in vitro mammalian cell proliferation.^37,38^ Recently, it was shown that aluminium (Al) surfaces possessing a random pattern of nanotextured ridges could inactivate SARS-CoV-2 within 6 h of exposure to the surface.^39^ Similar nanostructured Al surfaces exhibited antiviral activity toward respiratory syncytial virus (RSV) and rhinovirus.^40^ Nevertheless, the antiviral mechanism of the nanostructured surfaces remains to be understood.

In this study, our objective was to design and fabricate nanostructured silicon (Si) surfaces and evaluate their antiviral efficacy against hPIV-3. We present a systematic analysis of the antiviral effect of nanostructured silicon substrates on the inactivation of hPIV-3 viral particles. Plaque assay and Reverse transcription quantitative polymerase chain reaction (RT-qPCR) were conducted to assess virus viability after exposure to the nanostructured Si surfaces. Additionally, scanning electron microscopy (SEM), transmission electron microscopy (TEM), and cryogenic transmission electron microscopy (CryoTEM) were employed to investigate the overall morphology of hPIV-3. Finite element analysis was used to model the interaction of a virus particle with the nanostructured Si substratum, providing insights into the antiviral mechanism.

## Results and Discussion

### Fabrication and Characterization of Si Nanospike Surface

Recent studies have elucidated those geometrical parameters of a surface such as spacing and aspect ratio that are crucial for achieving maximum killing efficiency toward bacteria.^30,35,36,41^ Taking into consideration the size of the viral particles, we used reactive ion etching (RIE), which offers a straightforward process to achieve high aspect ratio, anisotropic structures and the ability to tune the height and spacing of the surface nanostructures by manipulating the etch time.^41,42^ Herein, inductively coupled plasma reactive ion etching (ICP-RIE) with etchant gases sulfur hexafluoride (SF_6_) and oxygen (O_2_) were used to etch a *p*-type boron-doped Si wafer for 20 min, resulting in the formation of sharp spikelike nanostructures with <100 nm interspacing.^43^ ICP-RIE involves using an inductively coupled plasma as the ion source of reactive species for etching.^44,45^ F^–^ ions are responsible for chemical etching of the Si surface, resulting in volatile SiF_*x*_ products. The SiF_*x*_ products, particularly SiF_4_, react with the O^–^ radicals, resulting in the deposition of a passivation layer of SiO_*x*_F_*x*_ on the Si substrate. Then, the bombardment of high-energy ions selectively removes this protective layer, exposing the Si surface to further etching by the F^–^ radicals. The competition between the removal and deposition of shielding material leads to the formation of fabricated structures with high aspect ratios. The nanotextured sample emerged darkened, appearing black due to their antireflective nature.

**Figure 1:**
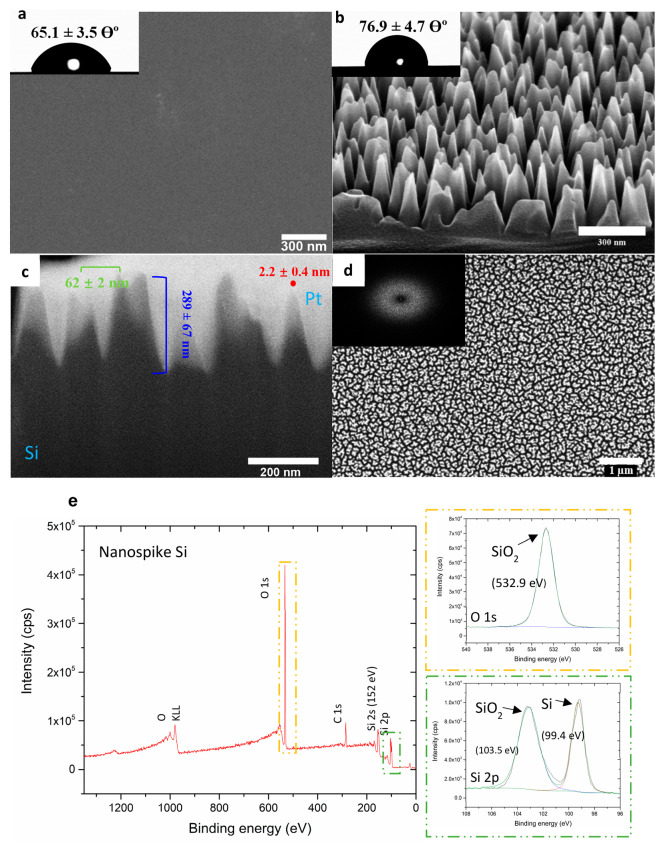
Representative SEM images of (a) the smooth Si wafer and (b) the fabricated nanospike Si surface. Inset images show the water contact angle measurements. Scale bars = 300 nm. (c) SEM images of the nanospike cross-section achieved by FIB milling. (d) SEM micrograph of the top surface of Si nanospike substratum. The inset shows an FFT image applied to top-view SEM images. (e) Wide scan XPS survey spectra of nanospike Si. The inset image depicts the deconvoluted high-resolution spectra of the O 1p (yellow box), with the binding energy of SiO_2_ being ∼532.9 eV and the Si 2p regions (green box), with the binding energy of SiO_2_ being ∼532.9 eV and that of Si being ∼99.4 eV.

Scanning electron microscopy (SEM) was used to characterize the Si nanofeatures’ geometrical parameters (spacing, nanospike height, cap diameter) (see Figures 1a–d). To measure the periodicity of the nanospikes, a two-dimensional (2D) fast Fourier transform (FFT) was applied to the top-view SEM image (Figure 1d). The interpillar (center–center) distance was estimated to be 62 ± 2 nm (Figure 1c). Focused ion beam (FIB) milling was used to investigate the cross-sectional profile of the nanospike surface. Tilt and cross-sectional micrographs (Figures 1b, c) revealed the spike height to be 289 ± 67 nm, and the tip diameter was estimated to be ∼1 - 2.2 nm. The wettability of nanospike Si was also determined via water contact angle (WCA) measurements (Figure 1a, b, insets) with surface energy (γ, mJ/m^2^) calculation (Table S1 in the Supporting Information) derived from it. Surface structuring did not dramatically influence the substratum hydrophilicity, as previously found;^36^ nanospike surfaces demonstrated a WCA of 76.9° (γ = 31.11 mJ/m^2^), whereas planar Si surfaces were 65° (γ = 64.23 mJ/m^2^), rendering both the nanospike Si surface and the planar control surface moderately hydrophilic. XPS analysis (Figure 1e, as well as Figure S1 in the Supporting Information) also showed no observable differences in surface chemistry between the planar and structured Si surfaces. Wide-scan XPS surveys (Figure 1e) showed peaks for O 1s, C 1s, Si 2s, and Si 2p. Deconvolution of the high-resolution O 1s and Si 2p regions confirmed the presence of a thin SiO_2_ surface passivation layer on both planar and nanostructured Si surfaces, confirming that ICP-RIE did not result in surface chemical changes.

### Nanostructured Surfaces Display Antiviral Activity against hPIV-3 by Inducing Envelope Deformation

**Figure 2:**
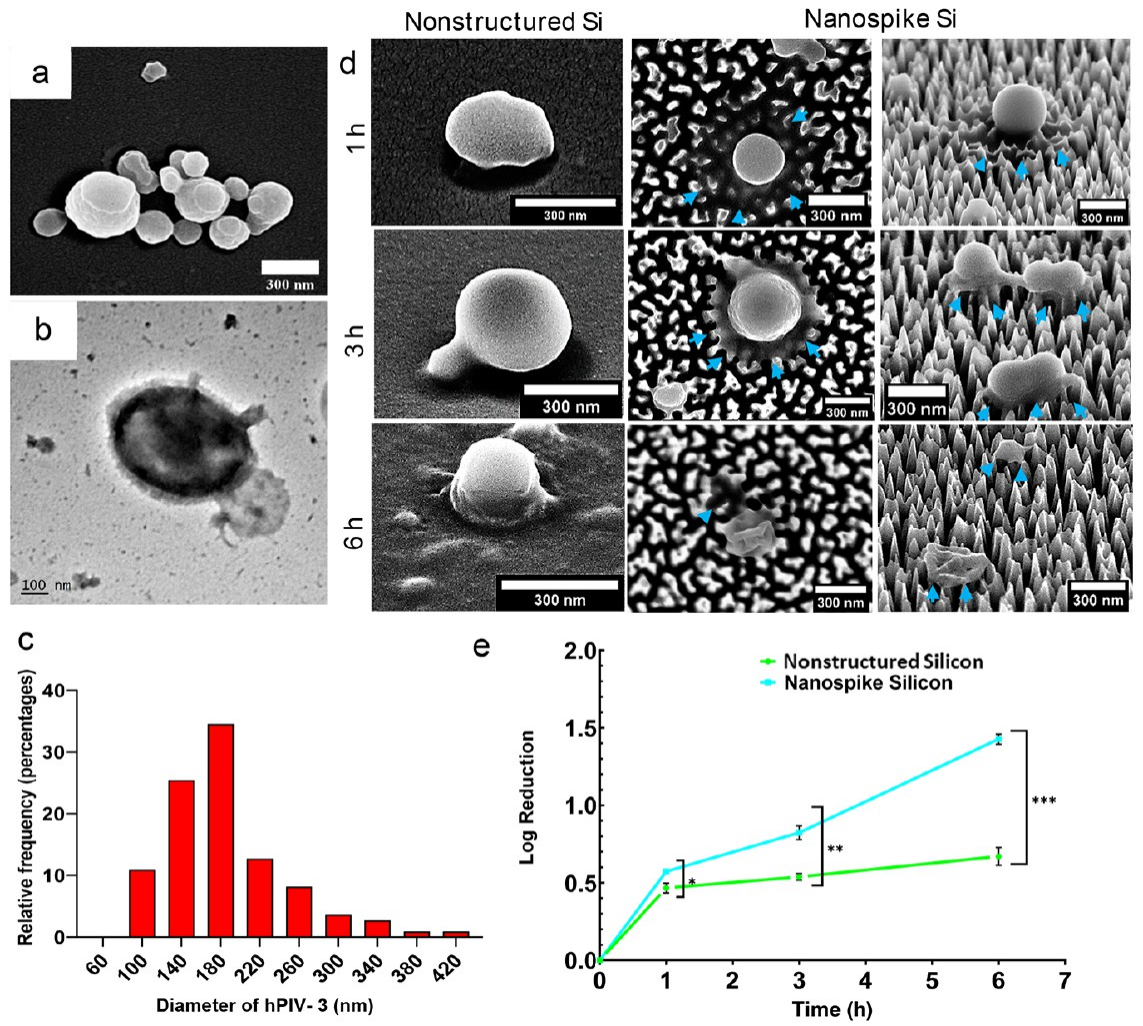
Interaction of hPIV-3 viral particles with silicon surfaces. (a) High-magnification SEM images of hPIV-3 fixed on nonstructured Si surfaces. (b) TEM micrograph of single hPIV-3 particle. (c) Histogram of the hPIV-3 sizes measured from SEM images. hPIV-3 exhibits a pleomorphic shape with a broad size distribution. Ranging between 100–420nm. (d) Representative SEM micrographs of hPIV-3 viral particles on nonstructured and nanospike (top and tilted view) Si surfaces. Spherical morphology typical for hPIV-3 was observed on the nonstructured surfaces. The morphology of hPIV-3 on nanostructured surfaces appeared to be altered as the viral particles were found to be deflated, losing particle integrity, which was seen following a 6 h incubation. The cyan arrows indicate a loss of cytosolic material and membrane deformation. (e) The dynamic inactivation of hPIV-3 over 6 h on nanostructured and nonstructured surfaces was measured using plaque assay expressed in terms of plaque forming units per mL (pfu/mL). The graph shows the log reductions (reduction of pfu/mL) of the hPIV-3 virus retrieved 1, 3, and 6 h after incubation. Results represent the mean ± standard deviation (SD) for three individual replicates from a single assay, representing three independent experiments (*n* = 3). Statistical differences are indicated as (*) *P* < 0.05, (**) *P* < 0.01, and (***) *P* < 0.001, calculated via Student’s *t*-test using GraphPad Prism (version 9.1.4).

hPIV-3 is a pleomorphic enveloped virus that belongs to the family *Paramyxoviridae*, group-V negative-sense single-stranded RNA viruses.^1^ SEM examination of hPIV-3 (Figures 2a, b) confirmed that the viral particles were mostly spherical, ranging between 100 and 420 nm in diameter; however, the majority (∼60%) were in the range of 140–220 nm (Figure 2c), which is in agreement with previously reported data.^1,46^ A broad range of virus shapes and sizes poses implications for assessing the virucidal activity of the nanospike surfaces; the variation in viral particle size may affect their interaction with the nanostructured surfaces and their overall resulting pathogenicity.

Surfaces incubated with hPIV-3 for 1, 3, and 6 h were examined under SEM to study the viral particle attachment and morphology (Figure 2d). The SEM micrographs showed that on nonstructured Si surfaces, the viral particles retained their typical rounded morphology following up to 6 h incubation. In contrast, the morphology of hPIV-3 particles on the nanostructured Si surfaces appeared compromised. In the tilted view of SEM images (Figure 2d, right panel), the membrane was stretched, and the sharp tips of the spikes penetrated and deformed the viral particles 1 and 3 h after incubation. Furthermore, following the 6 h of incubation, the viral particles were found to be deflated and exhibited a loss of particle integrity.

A plaque assay was performed to determine the hPIV-3 titer (representative of the number of infectious viruses) post-interaction with the nonstructured or nanostructured surfaces (Figure 2e, as well as Figure S2 in the Supporting Information).^47^ The data are expressed as the logarithmic reduction infectious viral titer: log reduction = , where *A* and *B* represent the number of viable viral particles (plaque forming units per mL) before and after the treatment.

A significant decline in infectious viral particles on nanospike Si at each time point, compared to nonstructured Si surfaces, is shown in Figure 2e. After 1 h of surface interaction, there was a 0.6 log reduction (74% drop) in infectious viral particles, compared to nonstructured Si surfaces; after 3 h, the reduction increased to a 0.8 log reduction (85% drop), and after 6 h, a 1.4 log reduction (96% drop) of infectious hPIV-3 virus was observed on the nanospike Si surface. RT-qPCR was used to monitor the copies of the viral RNA throughout the experiment. The RT-qPCR technique allows accurate quantification of the copies of the viral genomic RNA but does not discern between infectious or noninfectious virions (see Figures S3 and S7 in the Supporting Information). The number of hPIV-3 RNA copies appeared similar across all types of surfaces; statistical analysis showed no significant differences between the control, nonstructured Si, and the nanospike Si surfaces (*p* > 0.05). At 1 h, there were 5.8 log_10_ viral copies; at 3 h, there were 5.2 log_10_ viral copies; at 6 h, there were 4.2 and 4.0 log_10_ viral copies of hPIV-3 retrieved from the nonstructured and nanospike Si substratum, respectively. Cumulatively, these data indicated that the nanostructured Si surface disrupted hPIV-3 morphology, causing a loss of infectious potency, but the viral nucleic acid remains intact. While there is a reduction in infectious potency of the virus on planar Si surfaces, it is not to as significant an extent as that recorded for the Si nanospike array and the hPIV-3 morphology remained unchanged for the virus in suspension.

### Interfacial Interactions of hPIV-3 and Nanospike Si

Focused ion beam–scanning electron microscopy (FIB-SEM), TEM, and CryoTEM were used to investigate the virus–substratum biointerface further to assess the surface nanospike effect on the hPIV-3 envelope. The cross-sectional analysis allowed for visualization of the interface between a single viral particle and the nanospike topography.

**Figure 3:**
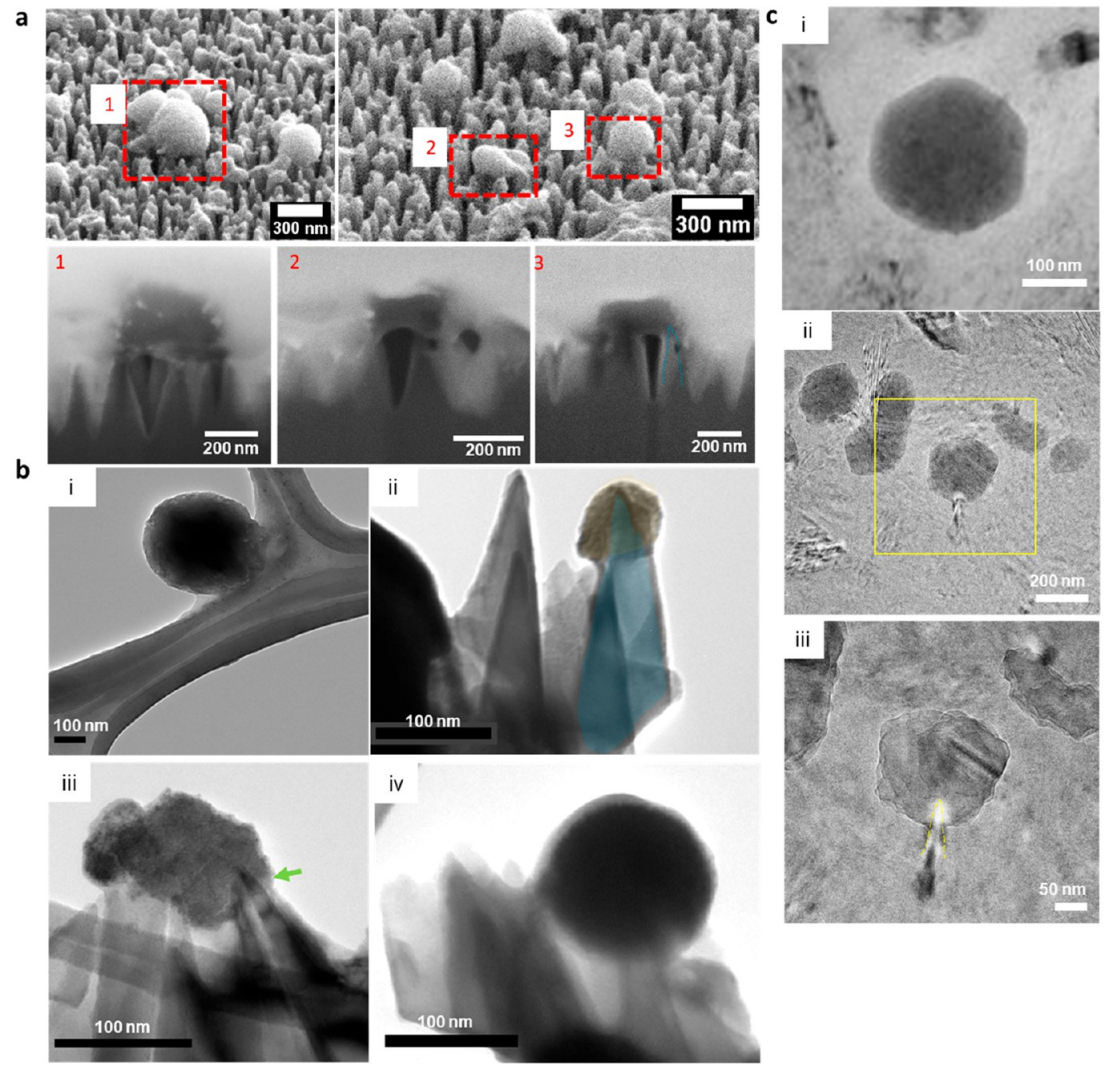
Mechanical disruption of viruses on nanospike Si surfaces. (a) FIB-SEM analysis of hPIV-3 on nanospike Si after 6 h of incubation. Cross-sectional analysis (bottom row) of each hPIV-3 particle was number coded correspondingly. Dotted lines highlight and trace the penetration of nanospike into hPIV-3. (b) TEM images of hPIV-3 particles and their interactions with surfaces ((i) TEM micrograph of a negatively stained hPIV-3 particle on lacey carbon films of copper grids after 6 h of incubation on a nonstructured Si surface; (ii–iv) typical TEM micrographs of hPIV-3 on nanospike Si after 6 h). The false-colored blue nanospike indicates the penetration depth into the false-colored yellow hPIV-3. The green arrow shows the site of insertion of the nanospikes. Scale bars = 100 nm. (c) CryoTEM images of hPIV-3 on nanospike Si surface after 6 h. Yellow dotted lines indicate indentation of the nanospikes into the virus.

Automated FIB milling was conducted on individual hPIV-3 particles attached to the nanospike Si to reveal whether the nanospikes had breached the viral envelopes. The FIB-SEM images revealed that 1–2.2-nm tip diameter nanospikes interacted with each viral particle. Typically, one-third of the spike tip penetrated the virus. These results were further confirmed by analysis of SEM, TEM, and CryoTEM micrographs which revealed that the nanospikes could penetrate the viral particles leading to deformation in the outer envelope (Figures 3b, c). Nanospikes discernibly extending toward the inside of hPIV-3 viral particles suggested their capability to pierce through the viral envelope. Such disturbance of the viral envelope may cause the 1.5 log reduction in infectious virus.

### Simulation of Virus–Nanospike Interactions

Our SEM and TEM experiments showed that the nanospikes on the Si substratum could pierce and rupture the encountering viral particle. We employed theoretical modeling to study the interactions between the virus and the Si nanospikes to gain a deeper understanding of this process. Specifically, we utilized the finite element method (FEM) through the COMSOL software, employing the contact method of COMSOL’s Structural Mechanics library.^48^ In the simulation, we represented the virus as a spherical object, while the nanospikes of the Si substratum were represented as a conical array. This allowed us to model their structural contact and investigate the forces involved during their interaction. The material properties assigned to the virus were as follows: Young’s modulus, *E* = 50 × 10^6^ Pa; Poisson ratio, ν = 0.3; and density, ρ = 1200 kg/m^3^.^49^ The material properties of the Si substratum are Young’s modulus *E* = 140 × 10^9^ Pa for the cones’ lower half and *E* = 60 × 10^9^ Pa for the upper half.^50^ The Poisson ratio of the cones is ν = 0.22, and the density is ρ = 2320 kg/m^3^. To compare with the experimental data obtained, the sphere in the simulation has a radius of 90 nm, while the cones used in the model have a height of 289 nm. The bottom and top radii of the cones are 52.5 and 5 nm, respectively. At the top of the cones is a hemisphere with a 5 nm radius to reduce the sharpness of the cone edges. The interpillar distance tip–tip of the cones is 62 nm (Figure 1c).

Consideration of the van der Waals forces, specifically the attractive term of the Lennard-Jones (LJ) potential, was used to consider the sphere (virus)–hemisphere (cone–tip) interaction.^51^ The LJ potential exhibits a square exponential decline starting at ∼0.3 nm, where the balance between the attractive and repulsive LJ potentials occurs.

The expression used to calculate the sphere-hemisphere attraction described by the LJ potential is *F*_vdw_ = , where *A* is the Hamaker constant for the studied interaction, that is in the order of 10^19^ J,^61^*R* is the radius of the hemisphere and has a value of 5 nm; and *d* denotes the distance between the points of interaction, which is varied during the simulation.

We established the upper surface of the spherical capsid as fixed, while the cone surface was subjected to a force directed toward the bottom of the sphere, normal to the cone tip. The force acting on the cone corresponds to *F*_vdw_, defined earlier as the van der Waals force.

At every point of the cone surface, the force is determined by the distance to the sphere along the cones' normal at that point. If the two surfaces are in contact, then the force becomes zero, representing the balance between the repulsive and attractive LJ potential at 0.3 nm.

**Figure 4:**
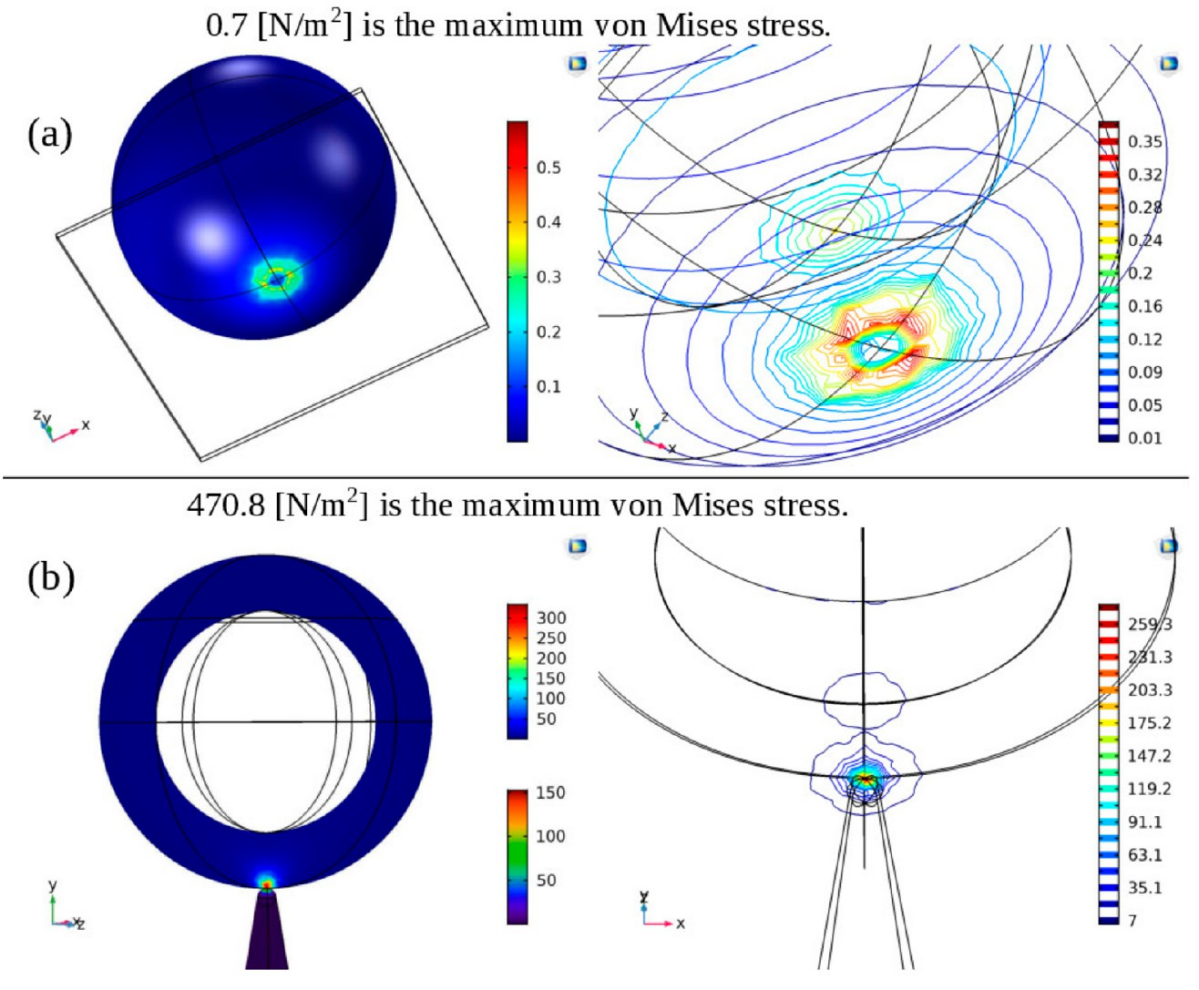
Interaction between a representative spherical virus object and a flat substrate, as well as a single nanocone, was modeled using the Finite Element Method (FEM) in COMSOL. In the case of the virus and the Si flat surface interaction (a), a typical stress formation resembling that of a spherical shell was observed. On the other hand, in the interaction between the virus and the Si single cone (b), an extensive stress concentration was observed at the region where the top of the cone and the virus meet.

To analyze the interaction between the virus and the nanospike Si substratum, we employed the stationary analysis of COMSOL’s contact method to define the equilibrium between the deformation of the two objects and their physical attraction. First, we defined the simpler interaction between a virus and a flat Si surface. As shown in Figure 4a, the virus envelope exhibited a circular stress pattern around the point of contact, like when a spherical shell is pressed from one side, as anticipated, confirming the validity of the simulation. Next, we investigated the interaction between a virus and a single Si nanocone. For the simulation between the virus and a single nanocone, an additional force was introduced in the case of a single pillar, acting on the lower hemisphere of the virus toward the cone along the *y*-direction. Although this force was 4 orders of magnitude smaller than the defined *F*_vdw_ force, it played a minor role in the overall interaction, primarily stabilizing the numerical method. Since the nanospike Si substratum comprises various geometrical features, incorporating this negligible external stabilization force helped to ensure numerical robustness.

Assuming that the virus envelope resembles a lipid bilayer, we can estimate the energy required to pierce and break through it using mechanical force. Based on a cone with a diameter of 1–2 nm, our calculations align closely with those for a 1-nm-diameter cylinder piercing a phospholipid membrane.^52^ Specifically, the membrane–nanospike interaction produces a vertical adhesion force that creates localized tension on the membrane that is maximal at the nanostructure tip due to its small surface area. Single-chain mean field (SCMF) calculations showed that the required energy to pierce the lipid bilayer is at least 100*kT*, making spontaneous piercing unlikely. Thus, an external force is necessary to achieve piercing. However, our recent simulations using SCFM showed that when the phospholipid bilayer is under tension, ultrasharp nanomaterials perpendicular to the membrane may spontaneously nucleate an unstable pore in the membrane and translocate.^53^

Based on the experimental evidence above, a viral particle attached to the nanospike Si substratum was most likely to interact with at least two nanocones. A noteworthy finding emerged when simulating the interaction between a virus and two cones. The configuration significantly depended on the distance between the cones. To explore this further, we simulated three different cases with interpillar distances of 62, 1.1, and 1.2 × 62 nm, representing distinct stress configurations on the virus. Similar to the case of the single cone, a negligible external stabilization force was added for each configuration.

**Figure 5:**
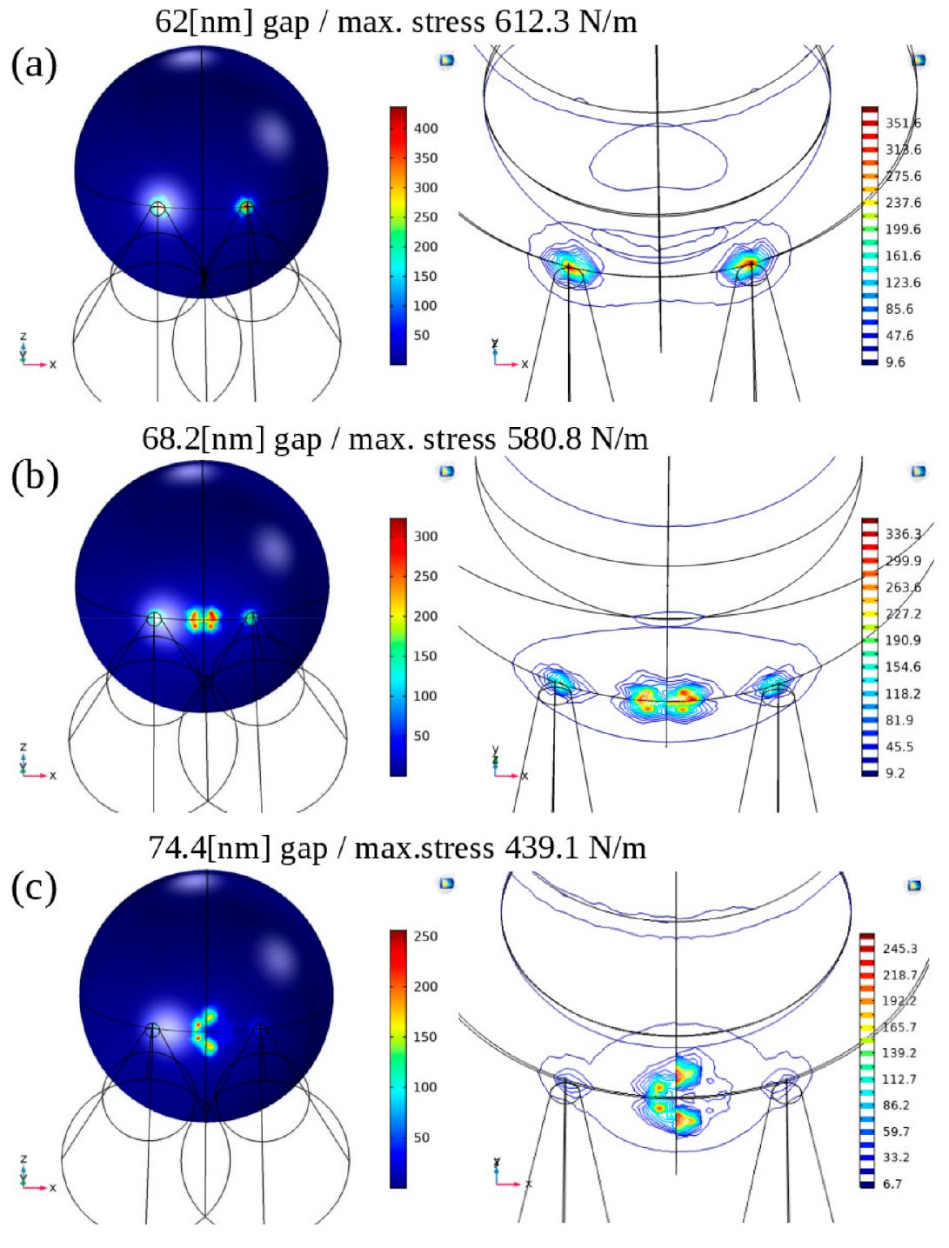
Finite element method (FEM) modeling performed using COMSOL of a spherical viral particle interacting with two nanocones. (a) When the nanocone spacing is 62 nm, the interaction between the virus and the two Si cones results in stress buildup primarily at the locations of the conical tips. (b) For a nanocone spacing of 1.1 × 62 nm, the virus and two Si-cones interaction led to stress buildup occurring in between the conical tips, while some stress remains at the tips. (c) With a nanocone spacing of 1.2 × 62 nm, the virus and two Si-cones interaction produce maximal stress buildup, primarily concentrated on the virus surface in between the conical tips.

In the case where the cone separation was 62 nm (Figure 5a), the maximum stress occurred at the top of the pillars. However, as the gap increased to 1.1 × 62 nm, stress began building up between the cones (see Figure 5b). Finally, in Figure 5c, we observed how the stress at the bottom of the virus envelope evolved when the gap increased to 1.2 × 62 nm. At this point, the maximum stress had already shifted away from the cone tips and was concentrated at the bottom of the virus envelope.

**Figure 6:**
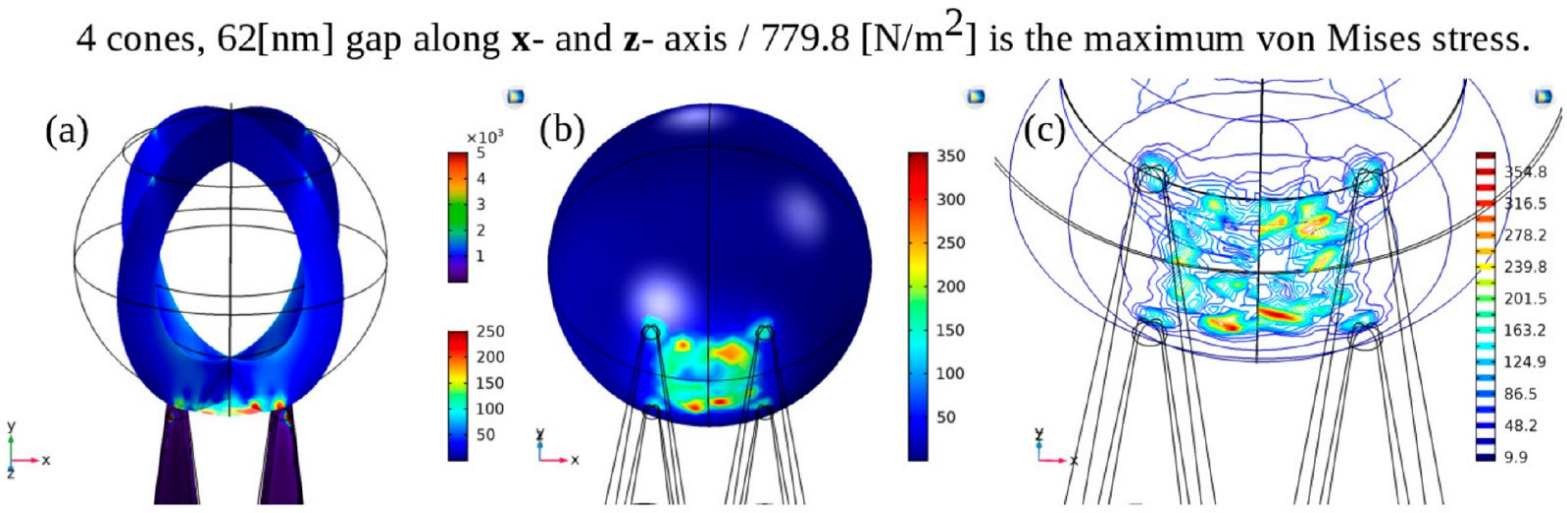
Finite element method (FEM) modeling using COMSOL of a spherical viral particle with four nanocones.(a) When the nanocone spacing is 62 nm, the interaction between the virus and the four Si cones results in extensive stress buildup primarily at the bottom of the virus. (b) 3D visualization of von Mises stress buildup at the bottom of the virus particle. (c) Heat map showing the accumulation of stress between the conical tips of the cones at the bottom of the virus. The maximum stress recorded was 779.8 N/m2.

In the case of a virus interacting with four cones, we observed the development of extensive stress at the bottom of the virus, as depicted in Figure 6. This finding aligns with the behavior observed in the case of two cones, where the distance between the cones increases sufficiently, such as in the scenario of a 1.1 × 62 nm gap.

To compare the stresses resulting from the different configurations and determine which one is the most destructive to the virus, we compiled the obtained stress values (Table 1).

**Table 1:** Comparison of Maximum Stress Distribution on 90 nm Spherical Viral Particles for Different Geometrical Configurations

configuration	resultant maximum stress on virus (N/m^2^)
planar	0.7
1 cone	470.8
2 cones (62 nm interpillar spacing)	612.3
2 cones (1.1 × 62 nm interpillar spacing)	580.8
2 cones (1.2 × 62 nm interpillar spacing)	439.1
4 cones (62 nm interpillar spacing)	779.8

The maximum values of Mises stress induced on the spherical viral particle in different nanospike geometries are highlighted in Table 1. It is evident that the flat surface induces the smallest maximum stress on the viral particle, followed by a single cone configuration, for a tensionless membrane. The case of two cones is particularly noteworthy, as it demonstrates that keeping the stress concentrated at the top of the cones results in higher maximum stress, albeit highly localized. However, when the distance between the cones increases, the stress distribution becomes more widespread, leading to a decrease in the maximum stress value. Nonetheless, virus that may have defects or are subject to external stress, which could potentially cause rupture.

The case of four cones is similar to the two-cone scenario since the distance between diagonally positioned cones is  nm, leading to stress distribution between the cones. However, the stress induced by the four cones is greater than that induced by the two cones. Therefore, we can expect that if we decrease the distance in between the cones and increase the number of cones, we can produce higher values of the stress. Also, with big number of cones that are not very close together, a large area of abnormal stress can be produced.

### Antiviral Nanospike Surfaces Retain Bactericidal Activity

**Figure 7:**
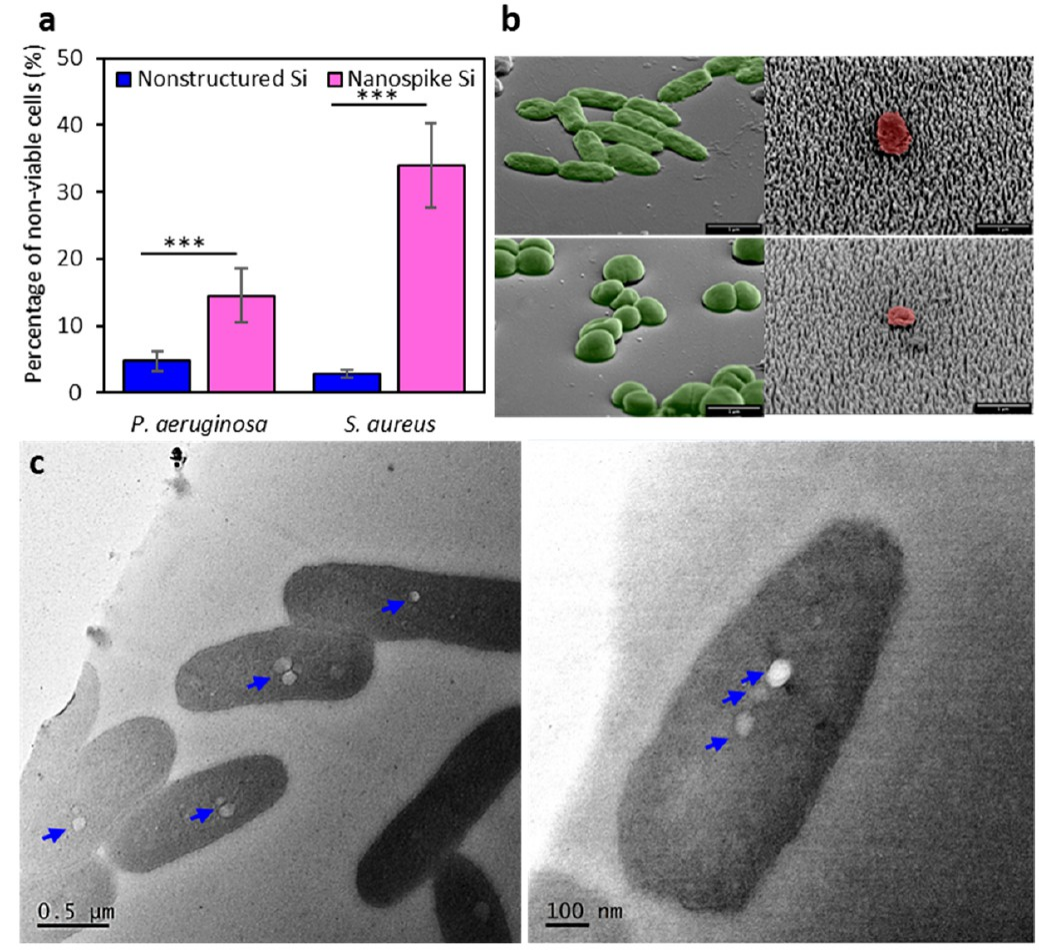
Bactericidal activity of nanospike Si surfaces against *P. aeruginosa* ATCC 9721 and *S. aureus* 65.8^T^ bacterial cells. (a) The percentage (%) of nonviable cells on the studied surfaces after 18 h of incubation. The proportion of nonviable *P. aeruginosa* and *S. aureus* was found to be 15% and 35%, respectively. The numbers of living and dead cells were calculated using the CLSM micrographs (*n* = 15). The series of three asterisks (***) implies statistical significance (*p* < 0.001). (b) Representative tilted SEM micrographs showing *P. aeroginosa* (top panel) and *S. aureus* (botom panel) bacterial cell attachment and morphology on nonstructured (left-hand side) and nanostructured surfaces (right-hand side). Live cells are coloured green and dead cells are coloured red. (c) Transmission electron microscopy (TEM) depicting the piercing of *P. aeruginosa* by nanospike Si. Nanospike-induced perforations are indicated by blue arrows.

To confirm whether the bactericidal efficiency of nanospike Si surfaces is also preserved on virucidal surfaces, the interactions of two pathogenic bacteria, *Pseudomonas aeruginosa* ATCC 9721 (Gram-negative) and *Staphylococcus aureus* 65.8^T^ (Gram-positive), were used. Examination of SEM micrographs of adhered bacterial cells on nanospike Si surfaces revealed that the cells of both bacterial strains were disrupted, compared to the cells attached to the control surface (see Figure 7b, as well as Figure S4 in the Supporting Information). In contrast, bacterial cells adhered to planar Si surfaces, retained their cellular integrity, and exhibited typical morphology without noticeable alterations.

Following examination of CLSM micrographs, the cell density and proportion of living and dead bacteria attached to the nanospike Si surfaces were reported (Figure 7a), and the CLSM micrographs revealed a significant decrease in bacterial cells adhered to the nanospike Si surface, compared to the control surface (Figure S4a, CLSM panel, in the Supporting Information). Specifically, *P. aeruginosa* and *S. aureus* adhered to the nanospike Si surface at 1.23 × 10^4^ and 4.80 × 10^3^, respectively. On the control Si surface, the total number of attached *P. aeruginosa* and *S. aureus* was assessed to be 9.26 × 10^4^ and 6.38 × 10^3^, respectively, significantly more than the number of attached cells on the nanospike Si surface. The estimated killing efficacy of nanospike Si surfaces against *P. aeruginosa* was 20%, while against *S. aureus*, it was up to 30%. The observed values demonstrated high statistical significance (*p* < 0.001), compared to those of the nonstructured Si surface, suggesting that, in addition to being virucidal, the nanospike Si surface is also bactericidal.

Resin embedding and ultramicrotome sectioning for TEM imaging was employed to investigate whether the nanospike Si could pierce bacterial cells, as we observed for viruses. Both *P. aeruginosa* and *S. aureus* were incubated on surfaces, followed by fixing and embedding in resin. The Si substratum was carefully detached from the resin, and the embedded cells were retained within the block. Analysis of the TEM images (Figure 7c) revealed spherical perforations in *P. aeruginosa* cells with sizes similar to the circumference of the nanospikes on the Si arrays. Notably, the size and number of perforations with bacteria differed, indicating different stages of spike–bacterial interactions, with larger holes resulting from deeper piercing, considering the somewhat conical shape of the spikes. However, a similar scenario was not observed in *S. aureus* cells (Figure S4c in the Supporting Information), potentially due to the structural characteristics of their more turgid cell wall.^54,55^

During the peak of SARS-CoV-2, worldwide concern over indirect contact transmission of viruses via fomites led to an extensive use of chemical disinfectants on high-traffic surfaces. In contrast, nanotextured surfaces offer a nontoxic and promising approach to combat surface viral contamination. However, the underlying antiviral mechanisms of these inert nanotextured surfaces remain unclear.

Here, using a combination of SEM, FIB-SEM, TEM, cryo-TEM, viral assays, and modeling, we investigated the effects of nonstructured and nanostructured Si surfaces on the hPIV-3 virus and derived the antiviral mechanism of action. Our results demonstrated that the nanospike-Si surfaces induced time-dependent viral inactivation. Gravity has a negligible effect on a single viral particle due to its mass of ∼1 fg (10^–15^ g) and aqueous solution providing buoyancy.^56^ Since hPIV-3 viral particles are suspended in liquid, the interaction between viruses and the substratum is largely influenced by Brownian motion.^57,58^ Furthermore, qRT-PCR showed no significant difference between the number of viral copies retrieved from Si and nanospike Si (at 1, 3, and 6 h), even though there is diminished infectivity shown in the plaque assay (Figure 2); implying the absence of chemical/physical reactions against the genetic material and supports the belief that the main cause of infectivity loss may be due to the impaired viral envelope (altered morphology) of hPIV-3 by the nanostructures on nanospike Si. Indeed, SEM and TEM imaging corroborated our hypothesis of mechanical inactivation of hPIV-3 particles as we observed a virus with altered morphology after attachment to the nanostructures, compared to the virus attached on smooth surfaces.

The rate of viral inactivation on nanostructured surfaces could be surface-nanopattern-dependent and viral-strain-specific. For example, in a similar study utilizing nanostructured aluminum surfaces, SARS-CoV-2 was inactivated more quickly than RSV and rhinovirus (RV) (Table 2). According to their data, the infectivity of RSV (RSV shares a similar family (*Paramyxoviridae*) to hPIV-3) appears to have undergone a 1 log reduction at 6 h while a more recent study involving nanostructured titanium surfaces, known for their potential to generate reactive oxygen species (ROS), managed to achieve a 2.6 log reduction at 5 h.^59^ The mechano-bactericidal effect of nanostructured surfaces toward bacteria was determined to result from a series of mechanical stress caused by, initially, the intrinsic attachment of the bacterial cells onto the sharp nanoprotrusions at multiple points, stretching the outer membrane until rupture.^29,60,61^ However, the mechanism of nanostructured surfaces’ antiviral action toward particles smaller than 1 μm, such as viruses, has not been studied thoroughly. Although we have previously speculated that the viruses could be disrupted physically if the nanofeatures are sized appropriately, *i.e*., the spacing between nanoprotrusions is small enough that the virus is not falling between the nanofeatures.^11^ In the case of mechanical inactivation observed with nanospike Si, we propose distinctly different virucidal actions, depending on whether the virus interacts with one, two, or four spikes. In the case of a single nanospike, we propose a piercing mechanism based on our observations from FIB-SEM and TEM images. According to our COMSOL simulations, in the case of spherical shapes, the contact area between the nanoprotrusions and the spherical surface is limited to specific regions. The reduced contact area results in a relatively smaller overall force being exerted on the spherical structure. In the case of a single nanocone of 1-nm tip diameter interacting with a virus, an external force was required to overcome the interaction energy barrier (∼100 *kT*) to result in envelope piercing. However, the viral envelope was modeled as a tensionless elastic lipid bilayer whereas the surface of the hPIV-3 envelope has been determined to be covered in a dense layer of glycoprotein^62^ and the envelope is known to undergo change in tension upon attachment to surfaces. Previous atomic force microscopy (AFM) studies have estimated that the external force required to pierce a Gram-negative bacterium is on the order of a few nN.^63^ However, membrane rupture is more likely to occur in stiff membranes, which tend to deform less and, thus, experience a higher concentration of stress at the tip apex, requiring less force to be penetrated. Indeed, the Young’s modulus of a Gram-negative bacterial membrane is in the order of 10–100 MPa, whereas the Young’s modulus of a viral capsid is orders of magnitude stiffer, in the range of 1–3 GPa.^64^ In our case, the sharp tip of the cone combined with the extreme stiffness of the membrane aids in the piercing process. Additionally, while the surface interaction is assumed to be attractive, governed by van der Waals forces, amino acid groups of proteins present in the membrane such as −NH_2_, −NH_3_^+^, −COOH, and COO drive adsorption onto the surfaces through double electrostatic interactions between the virions-ionized surface-active species and the oppositely charged surfaces, as well as hydrogen bonding (at neutral pH, most viral particles have a net negative charge, because they have an isoelectric point below 7^65^), increasing membrane tension. In the presence of a thin film of water, virus particles can establish a much stronger surface adhesion through hydrogen bonding between OH groups at the surface and the virus surface proteins.^65^ The adhesion force of a virion (MS2 coliphage) to a nanostructured Si surface was measured by using AFM to be 3.3 nN (5.2 nN for a smooth Si surface), which is larger than the force required to rupture a Gram-negative bacterium.^66,67^ In other work, the free energy of the interfacial interaction of enveloped human respiratory synctial virus (RSV; very closely related to hPIV-3) to silica surfaces was estimated to be 5.5 ± 0.4 mJ m^–2^, a net attractive interfacial interaction.^68^ Experimental data obtained by using quartz crystal microbalance (QCM) confirmed that the efficiency of their attachment to surfaces is highly correlated to surface free energy of interfacial interaction. In our study, virus adhesion to nonstructured and nanospike Si substratum was comparable, as determined from quantification of viral copies by RT-qPCR. Thus, the observed mechanical inactivation is likely attributed to the piercing effect of the nanospike Si structures. A similar piercing effect was observed as well in TEM images of *P. aeruginosa* bacterial cells (Figure 5). In the case of the virus interacting with more than two nanospikes, the COMSOL simulations revealed that the induced stress was relocated to the area on the membrane between the nanospike tips, rather than being localized at the tip apex. The resulting virucidal mechanism in this scenario would be similar to the mechano-bactericidal effect of nanostructured surfaces, whereby bacteria experience membrane stretching between each nanoprotrusion that overcomes the elasticity of the membrane, resulting in rupture.^29^

**Table 2:** Virucidal Activity of Nanostructured Surfaces

		Nanostructures Virucidal Efficiency				
virus	materials/patterns^a^	height (nm)	spacing (nm)	diameter (nm)	log reduction^b^	ref
human parainfluenza virus type-3 (hPIV-3)	silicon, nanospikes	289	60	4.9	1.4-log	this study
respiratory syncytial virus (RSV)	Al 6063, ridges	n/a^d^	250	161	1-log^c^	(40)
rhinovirus (RV)	Al 6063, ridges	n/a^d^	250	161	1.3-log^c^	
SARS-CoV-2	Al 6063, ridges	n/a^d^	250	161	5-log	(39)
SARS-CoV-2	Ti-6Al-4 V, nanosheets	300	n/a^d^	20	5-log	(69)
human coronavirus NL63 (HCoV-NL63)	Ti-6Al-4 V, nanosheets	300	n/a^d^	20	3-log	
human rhinovirus 16 (HRV-16)	Ti-6Al-4 V, nanosheets	300	n/a^d^	20	4-log	
respiratory syncytial virus (RSV)	Ti-6Al-4 V, nanosheets	298	n/a^d^	52	3.2-log	(59)

a All testing was droplet casting technique using 25 μL droplet in this study and 10 μL in refs (39), (40), and (69).

b Assessed against stock; 6 h incubation, this study and refs (39) and (40); 7 h incubation, refs (59) and (69).

c Data taken from ref (40).

d n/a = no data available.

These findings underscore the potential of nanotextured surfaces as a rapid and effective means of combatting viral contamination on high-touch surfaces.

## Conclusion

In summary, we fabricated nanostructured Si surfaces possessing a nanospike topography of 290 nm feature height and 60 nm pillar interspace. The surfaces demonstrated a significant 96% (1.4 log) reduction in viral infectivity at 6 h. The mechanism of viral inactivation is proposed to be physical disturbance of the viral particle integrity by the nanostructures toward the viral coat (envelope), which, in turn, leads to the loss of infectivity. The objective of our simulation was to compute the initial balance between spike stress and virus deformation due to the van der Waals interaction of their surfaces while ensuring that the viral particle remains below its plastic limit (avoiding large deformations). This analysis provided insights into how stress evolves, and which spike configurations could potentially damage the virus’ envelope. Overall, we observed that smaller gaps induce higher stress, which is mainly focused at the tips of the spikes. At certain separations, the stress starts to be distributed between the spikes, resulting in an abnormal stress in larger areas.

Viral shedding by humans via aerosol and droplets generally have low infectious doses and, hence, a 96% decrease of infectious particles in the contaminated surfaces would reduce the viral load enough to prevent disease in most healthy individuals.^70^ The combination of virucidal surfaces and common disinfectant practices will ensure >99.99% reduction of infectious viruses on common surfaces, helping the combat of surface viral transmission. In the future, further investigation of the variation in nanostructure geometric parameters on different materials toward different viruses will give insights into the design of effective, broad-spectrum antiviral surfaces. Our findings contribute to the advancement of surface engineering for antiviral and antibacterial applications. By understanding the virucidal mechanism of nanospike surfaces, our work greatly improves the continual development of innovative and efficient strategies to mitigate the spread of pathogens.

## Experimental Methods

### Nanofabrication

Nanospike Si surfaces were fabricated using *p*-type boron-doped 100 mm silicon wafers (University Wafer, Inc.) and processed with a Samco RIE-101iPH inductively coupled plasma (ICP)-assisted reactive ion etching tool. Silicon wafer surfaces were first cleaned sequentially with acetone, isopropyl alcohol, and water and then dried under nitrogen flow. Silicon wafers were then etched for 20 min according to the black Si recipe described elsewhere.^36^ Briefly, the etchant gases were SF_6_/O_2_ with respective flow rates of 35/45 sccm. Process pressure was 1 Pa, ICP power = 150 W, and an RIE bias power of 15 W was observed. After the etch process, the spontaneous passivation mask was removed using ultrasonication in a 10 wt % sulfuric acid solution. The etched surfaces have a black appearance due to their antireflective nature. As received, boron-doped 100 mm silicon wafers were used as control (nonstructured silicon) surfaces in all experiments. Before surface characterization and biological testing, all surfaces were cut into 1 cm pieces using a diamond pen (ProSciTech Pty Ltd., Kirwan, Queensland, Australia). Surfaces were sterilized by sonication in 70% ethanol (EtOH) (Chem-supply, Gillman, South Australia, Australia) for 15 min each. The surfaces were then delicately dried with a flow of nitrogen gas and stored in a desiccator to avoid moisture absorption until needed.

### X-ray photoelectron spectrometry (XPS)

Surface elemental analysis was performed on silicon (Si) and nanospike silicon (Nanospike Si) by using the Thermo Scientific K-alpha XPS instrument. This instrument employed a monochromatic X-ray source (Al Kα, *h*ν = 1486.6 eV) operating at 150 W. Photoelectrons were analyzed at a 90° angle to the surface, covering an area of 400 × 400 μm^2^. For the survey spectra, measurements were taken at 200 eV and recorded at a step size of 1.0 eV. Region spectra, on the other hand, were taken at 50 eV and recorded at a step size of 0.1 eV. To determine the relative atomic concentration of elements, XPS was used, and quantification was based on the peak area in the selected high-resolution region. Sensitivity factors specific to the instrument used were applied. During the analysis, scans were performed across the Si 2p, C 1s, and O 1s peaks to identify and quantify the presence of these elements on the surface.

### Surface Wettability

The WCA of the surfaces was measured by dispensing 5 μL of water on the surfaces and imaged using a Phoenix-MT(T) goniometer (SEO Co., Suwon, South Korea). Images were analyzed using ImageJ software (version 1.53e). The results were recorded as an average of five separate measurements for each substratum.

### Scanning Electron Microscopy (SEM)

Nanospike Si and nonstructured silicon surface samples were visualized at high resolution using a FEI Verios 460L XHR-SEM system operated at 5 kV. SEM image analysis. Image processing software, ImageJ (version 1.53e) were used to estimate the pillar characteristics, spatial distributions, spikes’ tip diameter, and degree of clustering (density/μm^2^). A fast Fourier transform (FFT) was applied to top-view SEM images to estimate the average pillar–pillar distance.

### Cell Culture

Vero (African green monkey kidney) cells (CCL-81, ATCC, Manassas, VA, USA) were maintained in Gibco Dulbecco’s Modified Eagle Medium (DMEM) (high glucose, l-glutamine, sodium pyruvate) supplemented with 10% heat-inactivated fetal bovine serum (FBS) supplemented with 1% penicillin/streptomycin. Cells were incubated at 37 °C in a humidified incubator supplemented with 5% CO_2_.

### hPIV-3 Propagation

hPIV-3 (Victorian Infectious Diseases Reference Laboratory Clinical Strain No. 93146859) was propagated in Vero cells. Vero cells (70%–75% confluency) were infected with hPIV-3 at a multiplicity of infection (MOI) of 0.1. The cells were inspected daily for cytopathic effects, and the virus-containing culture supernatant was harvested once 10%–20% of the cells were nonviable or when 50%–100% of the monolayer has fused to form syncytia (typically 3–5 days). The virus-containing supernatant was clarified from cell debris by centrifugation (3000 rcf for 15 min at 4 °C) and stored at −80 °C.

### hPIV-3 Purification

For imaging, hPIV-3 supernatant (from infected Vero cells) was freshly clarified and filtered using a 0.45 μm Millipore Millex Syringe filter. Virus was loaded onto a 20% (w/v) sucrose cushion in cold PBS at pH 7.4 and centrifuged at 100,000 rcf for 1 h, 30 min at 4 °C without brake for deceleration using a 369650 SW 32 Ti Swinging-Bucket Rotor (Beckman Coulter). Virus-containing pellets were resuspended in PBS at 4 °C for SEM analysis. Alternatively, the pellet was resuspended in a 0.2 M sodium cacodylate buffer for TEM analysis. Purified virus stocks were stored at −80 °C or kept at 4 °C for subsequent analysis.

### Incubation of Viral Particles with Nanostructured Surfaces

A 25 μL droplet of nonpurified hPIV-3 viral suspension (∼1 × 10^6^ pfu/mL) was deposited onto the nanostructured silicon and plain silicon wafers as positive controls, while viral maintenance media was deposited on silicon wafers as negative controls. The samples, including controls, were incubated in an ambient environment in darkness for 1, 3, and 6 h. After incubation, the viral suspension was retrieved, and the samples and controls were washed using cold PBS (25 μL) for 10 min in an ambient environment. The wash solutions were then retrieved and combined with the retrieved viral suspension. The combined sample was tested via the plaque assay and qRT-PCR.

### Plaque Assay

12-well were seeded with 2.5 × 10^5^ Vero cells and grown overnight prior to infection. Serially diluted viral inoculum was added and incubated at 37 °C and 5% CO_2_, with gentle agitation every 10–15 min. After 1 h, the inoculum was removed and replaced with 2.5 mL of agarose mix (1.8% SeaPlaque low melting temperature agarose [Bioscience Lonza] + Gibco’s complete DMEM supplemented with 1% antibiotic and 3.75 mg/mL fungizone). The plates were incubated at 37 °C with 5% CO_2_ for 7 days before being fixed with 2% formaldehyde overnight at room temperature (RT). The agar overlay was removed using a spatula and stained with 1% Crystal Violet for 1 h before rinsing with tap water. The plates were air-dried overnight, and the viral plaques were counted to determine the plaque-forming units (pfu)/mL.

### Quantitative Reverse Transcription Polymerase Chain Reaction (qRT-PCR)

Viral RNA was extracted from hPIV-3 supernatants from infected Vero cell cultures using the Isolate II RNA extraction kit (BIO-52072, Bioline, London, U.K.). Extracted RNA was used to generate a standard curve using qRT-PCR as previously described.^200^ The absolute number of RNA copies in sample supernatants was determined by extrapolation of the standard curve generated. Reduction of the viral RNA copy numbers was determined by calculating log_10_ (*C*_t_^T^*C*_t_^0^)/*k*, where *C*_t_^T^ is the threshold crossing value (*C*_t_) of viral RNA of a treated sample; *C*_t_^0^ is the initial *C*_t_ value of viral RNA of an untreated sample, and *k* is the slope of the linear regression for *C*_t_ value versus the logarithm of the viral RNA copy numbers. Statistics were calculated via a Student’s *t*-test using GraphPad Prism (version 9.4.1).

### SEM for Viral Morphology and Attachment

After retrieving virus inoculum from the nanostructured and nonstructured samples, the surfaces with the attached virus were fixed with 25 μL of fixatives (2.5% glutaraldehyde + 4% paraformaldehyde (1:1) mix, prepared with 0.1 M phosphate buffer) at RT (ca. 23 °C) for 1.5 h, followed by 25 μL of 2% osmium tetroxide (Electron Microscopy Sciences, Hatfield, PA, USA) fixation for 1.5 h at RT (ca. 23 °C). The surfaces were then washed three times with milli-Q water (Millipore), followed by a series of ethanol dehydrations (30%, 50%, 70%, 90%, and 100% × 2) with 10 min for each ethanol concentration. The samples were air-dried overnight. The dried samples were then sputter-coated with 7 nm iridium (Ir) using an EM ACE600 sputter coater (Leica, Germany). SEM images were obtained using an FEI Verios SEM instrument under high vacuum at an accelerating voltage of 5–10 kV.

### Measurement of hPIV-3 Sizes

Measurements for the diameter of hPIV-3 were carried out in ImageJ (version 1.53e) using at least 10 separate areas, measuring at least 100 individual viral particles as the whole population dataset. This dataset was then used for frequency distribution analysis using GraphPad Prism (version 9.4.1), and relative frequency (in terms of percentage) against diameter of hPIV-3 was plotted. Bin interval was set to 40 with the first and last bins set to 60 and 420, respectively.

### Focused-Ion Beam Milling–Scanning Electron Microscopy (FIB-SEM)

After retrieving virus inoculum from the nanostructured and nonstructured samples, the surfaces with the attached virus were fixed with 25 μL of fixatives (2.5% glutaraldehyde + 4% paraformaldehyde (1:1) mix, prepared with 0.1 M phosphate buffer) for 1.5 h at RT or overnight at 4 °C. Then, the samples were washed three times with 0.1 M cacodylate buffer followed by secondary fixation of 2% osmium tetroxide +1.5% potassium ferrocyanide mix for 2 h at RT. Then the samples were rinsed three times with milli-Q water. To prepare the samples for imaging, they were incubated with freshly made 1% thiocarbohydrazide (THC) for 20 min as a mordant, followed by a second staining using 2% osmium tetroxide for 30 min. The samples were then dehydrated with a series of ice-cold ethanol dehydrations (20%, 50%, 70%, 80%, 90% and 100% × 2), 10 min for each ethanol concentration. After air drying for 20 min, the surfaces were then sputtered-coated with 10 nm Ir. The surface samples were milled and visualized using FEI Scios Dual Beam FIB-SEM. To protect the sample surfaces during milling, a platinum protection layer was applied by using an e-beam Pt deposition process. Cross-sectional milling processes were implemented.

### Transmission Electron Microscopy

An aliquot of 20 μL of purified and concentrated hPIV-3 (resuspended with 0.1 M cacodylate buffer) was drop-casted on strong carbon grids (GSFC200CU-SA, ProSciTech Pty, Ltd.) for 20 min at RT before they were negatively stained with 1% uranyl acetate (Electron Microscopy Science, Hatfield, PA, USA) for 1 min and then washed twice with milli-Q water. The carbon grid was then air-dried on filter paper overnight in a sterile environment. The carbon grid was then viewed under TEM (Model JEM 1010. JEOL USA, Inc., Peabody, MA, USA) at an accelerating voltage of 100 kV.

For viewing viruses on surfaces, a similar sample preparation method for FIB milling was utilized. However, after the series of ethanol dehydrations, samples were kept in 100% ethanol before being gently scraped with a sterile disposable scalpel (Swann-Morton Limited, Sheffield, England) in one direction. An aliquot of 20 μL of 100% ethanol was used to resuspend the scrapings before drop-casting them onto a lacey carbon grid (GSLC205-CU, ProSciTech Pty, Ltd.). The grid was air-dried for 3 h before being viewed via TEM under the conditions described above.

### CryoTEM

After fixation and staining with 2% osmium tetroxide, samples were stored in milli-Q water before gently scraping the surface with attached virus using a sterile disposable scalpel (Swann-Morton Limited, Sheffield, England) in one direction. An aliquot of 20 μL of milli-Q water was used to resuspend the scrapes in a microcentrifuge tube. Cryogrids were prepared by plunge freezing 3 μL of applied virus-Si nanospike suspension following a blot time of 15 s using a Leica EMPG-2 vitrobot. Cryogrids were viewed using a Tecnai F30 at an accelerating voltage of 200 kV.

### COMSOL Simulations

The contact method of COMSOL was employed in a stationary analysis to determine the equilibrium between the deformation of the spherical virus and the conical spikes of the Si surface when the cones interact with the virus (refer to the *F*_vdw_ equation above). This modeling utilizes the contact method available in COMSOL’s Structural Mechanics library.^48^ For the contact analysis, we adopted the default Penalty Method, wherein the contact pressure penalty factor represents the stiffness of a spring positioned between the boundaries of the contact pair, i.e., the virus, and the substrate surface. The value of the penalty factor used was calculated as the Young’s modulus divided by the minimum element size of the spherical body representing the virus.

Considering the sizes and shapes of the objects, we determined the appropriate implementation of meshing restrictions for COMSOL’s contact method. For further details, refer to the “Contact Analysis” section in ref (48).

**Figure 8:**
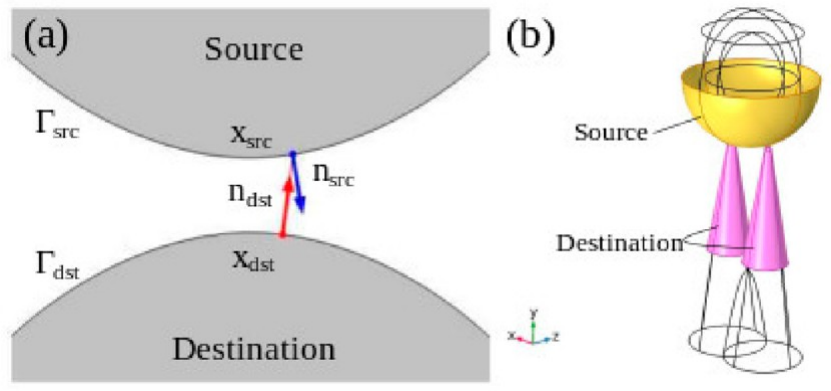
Schematic of the contact method of COMSOL. Panel (a) shows the source and destination surfaces, normal, and gap distance. Panel (b) shows the corresponding source and destination contact surfaces (denoted in yellow and purple, respectively).

**Figure 9:**
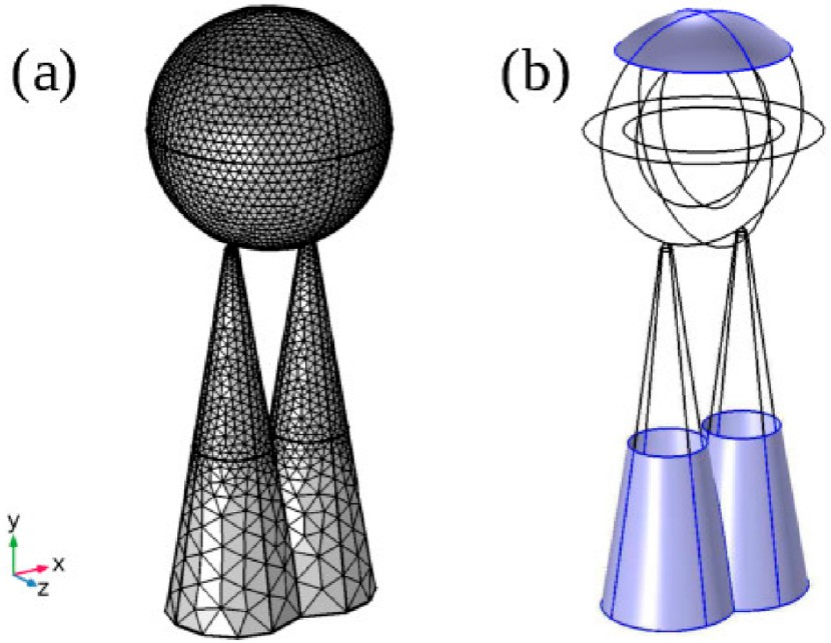
(a) The mesh of the sphere–cone model. The sphere has constant size of 8 nm while the cones’ elements increase from 3 nm at the tips to 8 nm at the bases of the unconstrained parts of the cones. (b) The surfaces that have constrains. The upper blue surface of the sphere is fixed in space. The lower blue surface of the bases of the cones is restricted along the *x*- and *z*-axes.

In short, the method is as follows: two boundaries, source, and destination were defined (see Figure 8). For the definition of the distance between the surfaces (Γ_src_ and Γ_dst_) that are going to be in contact, COMSOL uses the following operator:
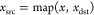


This way, for each point *x*_dst_ on the destination boundary Γ_dst_, point *x*_src_ is defined as the nearest intersection of the source boundary Γ_src_ and the destination normal *n*_dst_. Then, the important quantity of distance between the source and destination boundary (*g*_geom_), or gap function, is set. Note that *g*_geom_ is not necessarily the closest distance between the two boundaries:
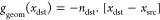


A positive gap infers a geometrical separation between the points, while a negative gap implies an overclosure of Γ_src_ and Γ_dst_. Considering that the points on the destination side connect to the source side via the destination’s normal (see Figure 8a), a coarse mesh on the destination side would miss an element on the source side and leave it without connection. Moreover, it is recommended that the element size of the destination’s mesh should be at least two times smaller than the source’s mesh. Additionally, the boundary of the stiffer object should be meshed coarser than that of the softer one. As a result, the source is typically set as the boundary of the stiffer object. In our simulation, we have two domains in contact: spheres and cones, with small hemispheres as tips. Naturally, we want the mesh to be finer for the smaller object, which are the cone tips. However, this contradicts the advice that the stiffer object should have a coarser mesh. In our model, the cones act as the destination and the sphere is the source. This arrangement allowed us to have triangular mesh elements on the sides of the spheres with a size of 8 nm, which is not excessively small. This is crucial for two reasons. First, it enabled us to create a cone tip with a smaller mesh, specifically triangles with sides of 3 nm (see Figure 9a). Second, the coarser mesh of the sphere introduces geometrical asymmetry or defects that resemble the property of geometrical defects in a hexagonally tiled spherical capsid (see ref (62)). Specifically, due to the inability to fully tile the sphere with hexagons, a few pentagon defects enable the shell to be enclosed as a sphere topologically. In our simulation, the sphere was tiled with triangles, and if the virus is meshed very finely, the asymmetric clusters on the sphere’s surface appeared smaller than the size of the pentagrams, where stress is expected to evolve on the capsid in reality. There are two shortcomings. First, the stiffer object has finer mesh which is not advised by the COMSOL manual (see ref (48)). Second, only the normal of the very top of the cone tips can reach the virus. Thus, the normal of the cones easily misses the virus, and the search function cannot reach a value of more than ∼40 nm. Therefore, when both surfaces are in contact, 40 nm is the maximum distance of interaction between the objects in that configuration, resulting in an interaction that is only affected by the cone tips. We complemented the model with a constant force that acts on the lower hemisphere of the virus and pulls its surface toward the base of the cones. It is equivalent to the van der Waals interaction of a particle 100 nm away from the surface and resembles the overall attraction to the substrate.

We implemented two restrictions, as illustrated in Figure 9b. The upper part of the virus boundary is fixed, while the surface of the cone bases cannot move laterally along the *x*- and *z*-axes. This allows the cone bases to move toward the virus along the *y*-direction, while their upper parts are free to bend or twist.

### Bacterial Growth Conditions and Experimental Setup

*Pseudomonas aeruginosa* ATCC 9721 and *Staphylococcus aureus* CIP^T^ 65.8 were derived from the American Type Culture Collection (ATCC, Manassas, VA, USA) and the Culture Collection of the Institut Pasteur (CIP, Paris, France), respectively. Bacterial stocks were recovered from 80 °C for 24 h on nutrient agar (Oxoid, Thermo Fisher Scientific, Waltham, MA, USA) at 37 °C. Before each experiment, bacterial strains were subcultured on nutrient agar until they reached the logarithmic growth phase (18 h). Freshly subcultured plates of *P. aeruginosa* and *S. aureus* were used to prepare a bacterial cell suspension in nutritional broth with an OD_600 nm_ of 0.1 (Oxoid - Thermo Fisher Scientific, Waltham, MA, USA). The surfaces were submerged in 1 mL of bacterial solution in a sterile 12-well plate (Corning, Merck Life Science Pty Ltd., Bayswater, Australia) for 18 h at 25 °C in darkness and under static circumstances. There were two duplicates of each surface type. Three independent replicates were used to corroborate the findings.

### Confocal Laser Scanning Microscopy (CLSM)

A Zeiss LSM 880 Airyscan upright CLSM (Carl Zeiss Microscopy, Oberkochen, Germany) operated with a 63× water-immersion objective (ZEISS 60×/1.0 VIS-IR) was used for visualization of *P. aeruginosa* and *S. aureus* attachment on surfaces after an 18 h incubation period. Prior CLSM, samples were gently washed with PBS as described elsewhere.^71^ Samples were then stained with the LIVE/DEAD BacLight Bacterial Viability Kit, L7012 (Molecular Probes, Invitrogen, Mt. Waverley, Australia). The BacLight Bacterial Viability Kit consists of a combination of the fluorescent nuclear stain SYTO 9 and propidium iodide (PI). SYTO 9 is a membrane-permeable green fluorescing dye that enables the measurement of total cells. PI is a red-fluorescing reagent that translocates only through a ruptured membrane. The total number of living and nonliving cells were determined by using CellC—a Matlab program.

### Bacterial Sample Preparation and SEM Analysis

For the visualization of the bacterial cell morphology, samples were fixed for 35 min in 2.5% glutaraldehyde. Following dehydration in a series of ethanol concentrations of 30%, 50%, 70%, 90%, and 100% (×2) for 15 min each, samples were sputter-coated with 7 nm of iridium (Ir). SEM images were obtained using an FEI Verios 460L XHR-SEM under high vacuum at an accelerating voltage of 3 kV.

### Resin Embedding and Sectioning

After the solution was incubated and fixed microorganisms (viral or bacterial) on surfaces, the surfaces were subjected to a series of ethanol dehydration steps (50%, 70%, 90%, 95%, and 100% × 2) for 15 min at each ethanol concentration. Then, 100% acetone was added twice for 15 min each time. Finally, the acetone was removed, and a 1:1 mix of 100% acetone: 100% Spurr’s resin (Electron Microscopy Sciences, Hatfield, PA, USA) was added. The surfaces (with the resin mix on top) were placed in a glass Petri dish (covered) and rotated (slow speed) on an Orbitron rotator (Thomas Scientific) overnight. The Orbitron rotator was covered with aluminum foil to prevent light exposure. The next day, fresh 1:1 acetone: Spurr’s resin mix was added onto the surfaces for 2 h with covers removed while still rotating on the Orbitron rotator. Then, the resin mix was removed, 100% fresh resin was added, and the surfaces were placed in a vacuum chamber for 2 h. The previous step was repeated another time before the samples were placed into a 70 °C oven for polymerization for at least 2 days. After polymerization was completed, the samples were first submerged in liquid nitrogen, and then, the substrates (entrapped in resin block) were detached using tweezers. The detached resin block was situated in a metal specimen holder for ultramicrotome sectioning. Thin serial sections of 60 nm (longitudinally and latitudinally) were acquired using an ultramicrotome equipped with a diamond knife. Sections were collected on lacey carbon grids and were post-stained with 2% uranyl acetate solution before TEM imaging with similar conditions described above.

### Statistical Analysis

All statistical analyses were performed using GraphPad Prism (version 9.4.1) and data were presented as mean ± SD, representative of three independent experimental replicates, performed in triplicate (*n* = 3) unless otherwise indicated. Student’s *t*-test was employed to determine the statistical difference of all antimicrobial bioassays.

## Supporting Information Available

Supplemental information includes various experiment-derived data such as additional XPS and surface energy measurements, RT-qPCR results, extra antibacterial analyses, the natural inactivation rate of tested hPIV-3, and droplet evaporation rates on surfaces; supplementary methods are also covered (PDF)
