# Supplementary material for: Piercing of the Human Parainfluenza Virus by Nanostructured Surfaces
Source: ACS Nano. 2023 Dec 21;18(2):1404–19. doi: 10.1021/acsnano.3c07099 (PMC10902884; doi:10.1021/acsnano.3c07099)
Supplement: Supplementary file 1 — nn3c07099_si_001.pdf [file nn3c07099_si_001.pdf]

Supporting information

Piercing of Human Parainfluenza Virus by Nanostructured Surfaces

Samson WL. Mah[1,2], Denver P. Linklater[1,3], Vassil Tzanov[4], Vladimir A. Baulin[4], Phuc H. Le[1], Chaitali Dekiwadia[5], Edwin Mayes[5], Ranya Simons[2], Daniel J. Eyckens[2], Graeme Moad[2], Soichiro Saita[6], Saulius Joudkazis[7], David A. Jans[8], Natalie A. Borg[9*], Elena P. Ivanova[1*]*

[1] School of Science, STEM College, RMIT University, Melbourne, Victoria 3000, Australia

[2] CSIRO Manufacturing, Clayton, Victoria 3168, Australia

[3] Department of Biomedical Engineering, Graeme Clarke Institute, The University of Melbourne, Parkville, Victoria 3010, Australia

[4] Departament de Química Física i Inorgànica, Universitat Rovira i Virgili, C/ Marcel.lí Domingo s/n, Tarragona 43007, Spain

[5] RMIT Microscopy and Microanalysis Facility, STEM College, RMIT University, Melbourne, Victoria 3000, Australia

[6] The KAITEKI Institute Inc., Chiyoda-ku, Tokyo 100-8251, Japan

[7] Optical Science Centre, Swinburne University of Technology, Hawthorn, Melbourne, VIC 3122, Australia

[8] Nuclear Signalling Laboratory, Department of Biochemistry and Molecular Biology, Monash University, Monash, Victoria 3800, Australia

[9] School of Health and Biomedical Sciences, RMIT University, Bundoora, Victoria 3083, Australia

Additional XPS Measurements

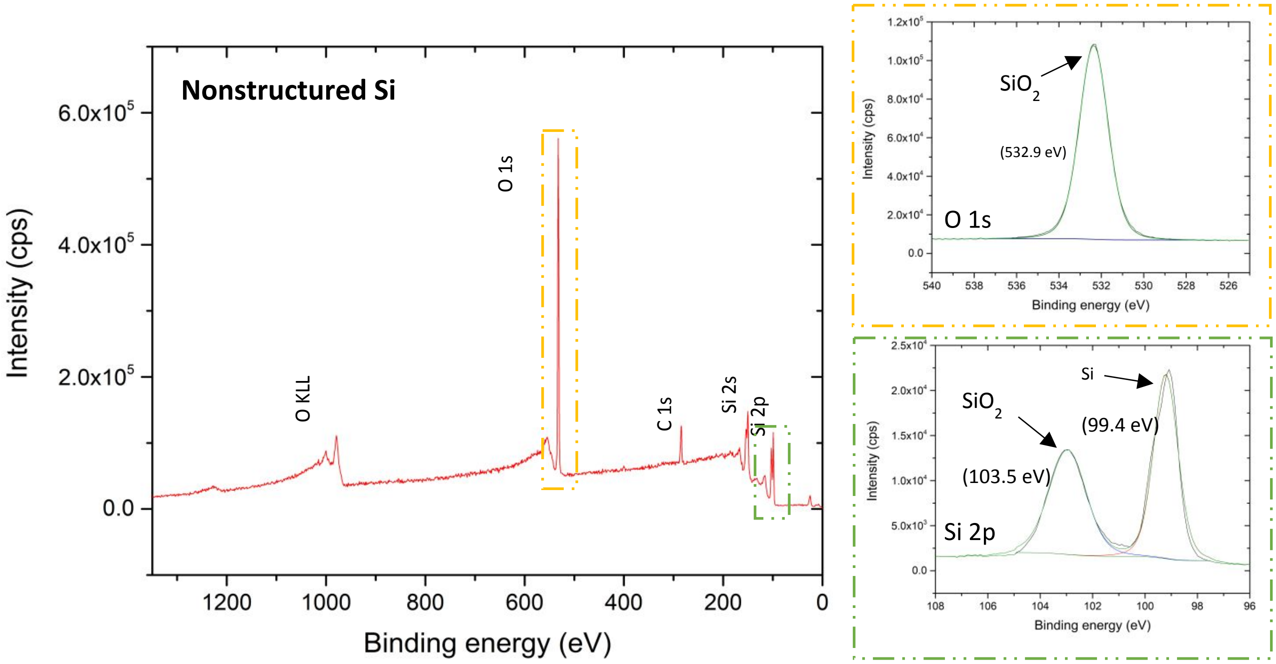

Figure S1. Wide scan XPS survey spectra of non-structured Si. The inset images depict the deconvoluted high-resolution spectra of the O 1p (yellow box) and the Si 2p regions (green box).

Surface Energy of Si and Nanospike Si

Table S1 below shows the contact angles on various surfaces and the corresponding surface tension values for non-structured Si and nanospike Si as well as the calculated total surface energy.

Table S1: Surface Energy of Silicon Surfaces

**Surface Type**	**Water Contact Angle (θw)**	**Ethylene Glycol Contact Angle (θeg)**	**Polar Component (γp) (mJ/m²)**	**Dispersion Component (γd) (mJ/m²)**	**Total Surface Energy (γ) (mJ/m²)**
Non-structured Si	65.12°	44.0°	51.0	21.8	64.23
Nanospike Si	76.9°	2.67°	29.3	19.0	31.11

To calculate the surface energy of surfaces, the contact angles of two probing liquids (water and ethylene glycol) were measured (Table S1), from which the surface energy can be obtained on the basis of the geometric mean method (1) :

$$\gamma_l(1+\cos\theta) = 2(\gamma_s^d \gamma_l^d)^{1/2} + 2(\gamma_s^p \gamma_l^p)^{1/2} \quad (1)$$

In this method, the subscripts "l" and "s" are used to represent the liquid and solid phases, while the superscripts "d" and "p" denote the dispersion and polar components of the surface energy, respectively. The two probing liquids' dispersion and polar components were given as follows: $\gamma_w^d = 21.8$ mJ m^{-2} and $\gamma_w^p = 51.0$ mJ m^{-2} for water as well as $\gamma_{e-g}^d = 29.3$ mJ m^{-2} and $\gamma_{e-g}^p = 19$ mJ m^{-2} for ethylene glycol, see (2). The values are similar to previous findings of silicon surface energy of different methods albeit slightly higher (3, 4)

Antiviral activity measurements

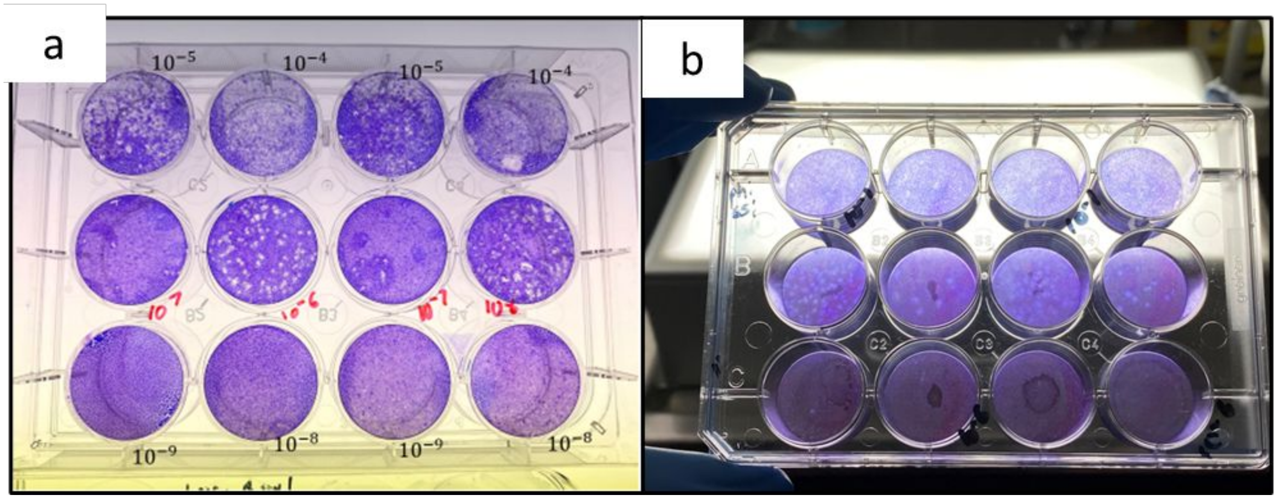

Figure S2. hPIV3 plaque overlay comparisons utilizing 12 well plates. Veros cells were plated at 2.5 x 10^5 cells in 12-well plates and infected with 200 µl serially diluted inoculum. After infection 2.5 ml overlays of 0.8% low melting agarose and 0.8 % growth media (final concentrations), were applied. Plaques were counted and titered. (a) hPIV-3 stock (b) nanospike silicon after 1h.

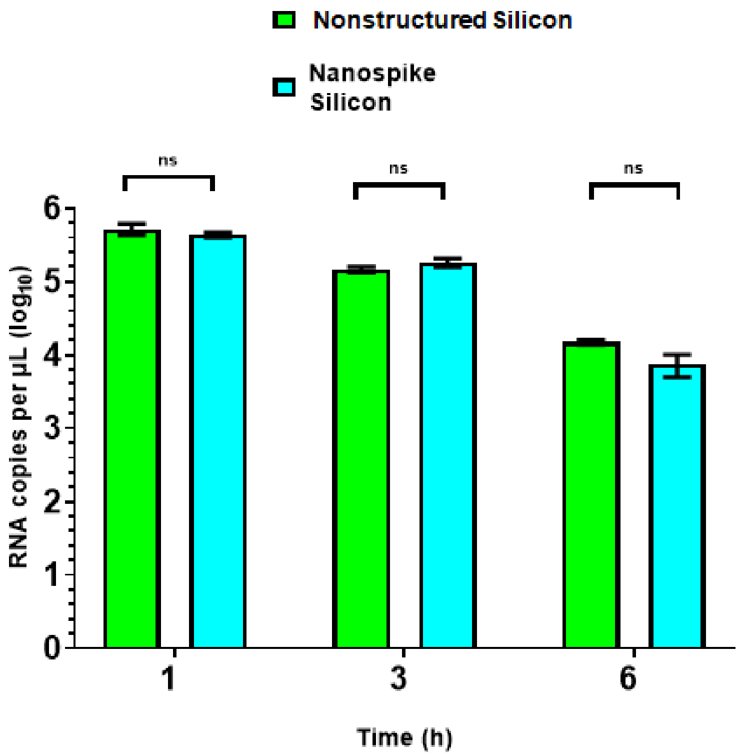

Figure S3. The number of genome copies in retrieved viral suspensions using reverse transcription quantitative polymerase chain reaction (RT-qPCR). The graph is showing log_{10} number of RNA copies per µL for hPIV-3 virus retrieved at 1, 3, and 6 h.

Bactericidal activity

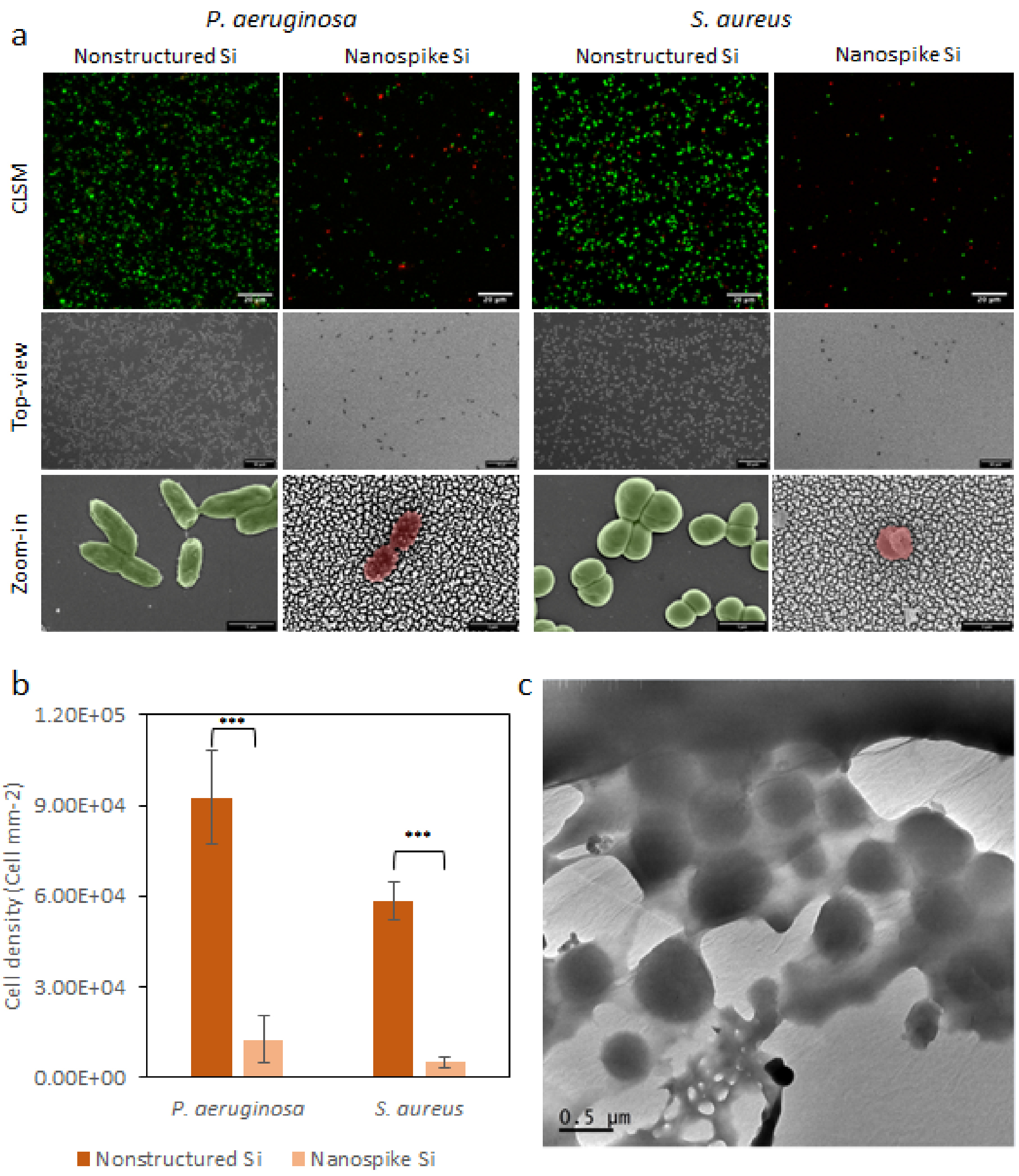

Figure S4. Bactericidal activity of nanospike Si surfaces against *P. aeruginosa* ATCC 9721 and *S. aureus* 65.8T bacterial cells. (a) Representative CLSM and SEM micrographs showing the bacterial cell attachment and morphology upon attachment on nonstructured and nanostructured

surfaces. (b) Comparative analysis of the density of attached cells and ratio (%) of non-viable cells on the studied surfaces after 18 h incubation. A substantial decrease of approximately 5-fold of the attached cells was observed on the nanostructured nanospike Si surfaces. *** implies statistical significance ($p < 0.001$). (c) TEM images of *S. aureus* after incubation on nanospike Si.

hPIV-3 natural inactivation rate

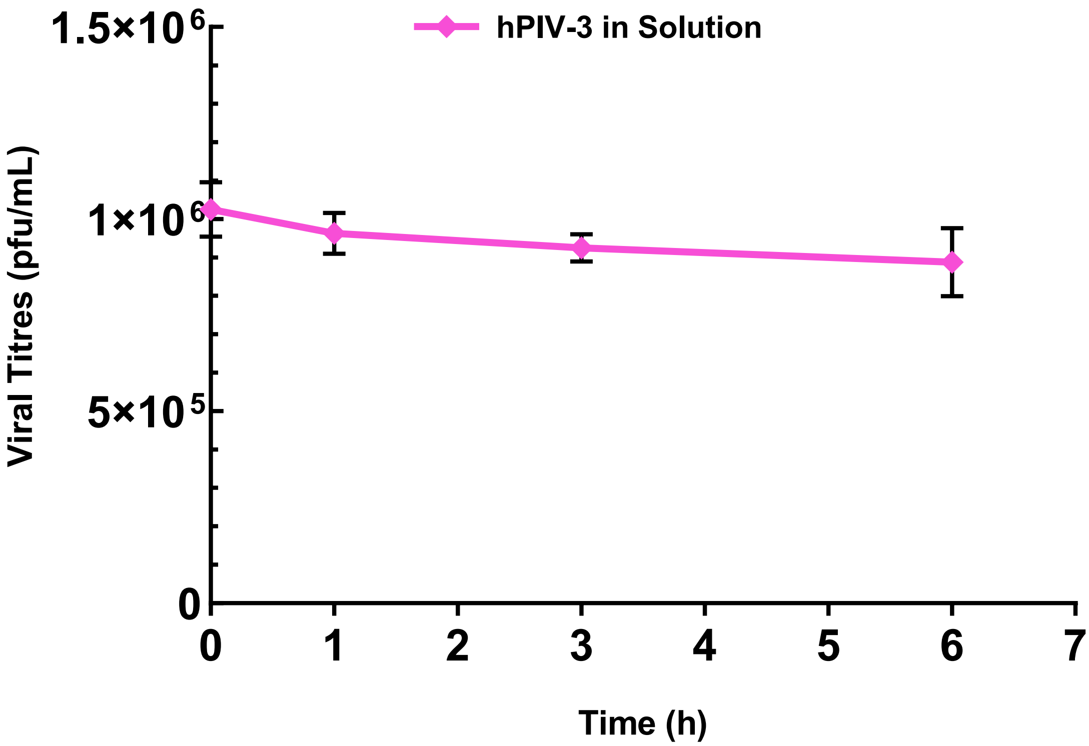

Figure S5. Number of infectious human parainfluenza virus type 3 (hPIV3-) particles retrieved at 1, 3 and 6 h from solution (viral maintenance medium). The graph is showing the number of plaques forming units (pfu/mL) for hPIV-3 virus retrieved at different time period. Error bars are

one standard deviation (n = 2). Statistical analysis showed no significant differences across each time point.

Desiccation rate of droplets on surfaces.

The amount of water evaporation was calculated over the course of a 12 h period, Figure S6. As shown, the 25 μL droplet was mostly evaporated on both surfaces by 12 h. During the desiccation process, water may form both an internal meniscus inside each virus particle and an external meniscus surrounding each particle. This would lead to the generation of forces that are in the range of tens of nanonewtons (5), which could be sufficient to deform or break the virus particles (6). Therefore, capillary forces may play an important role in the observed antiviral effects of the nanostructured surface.

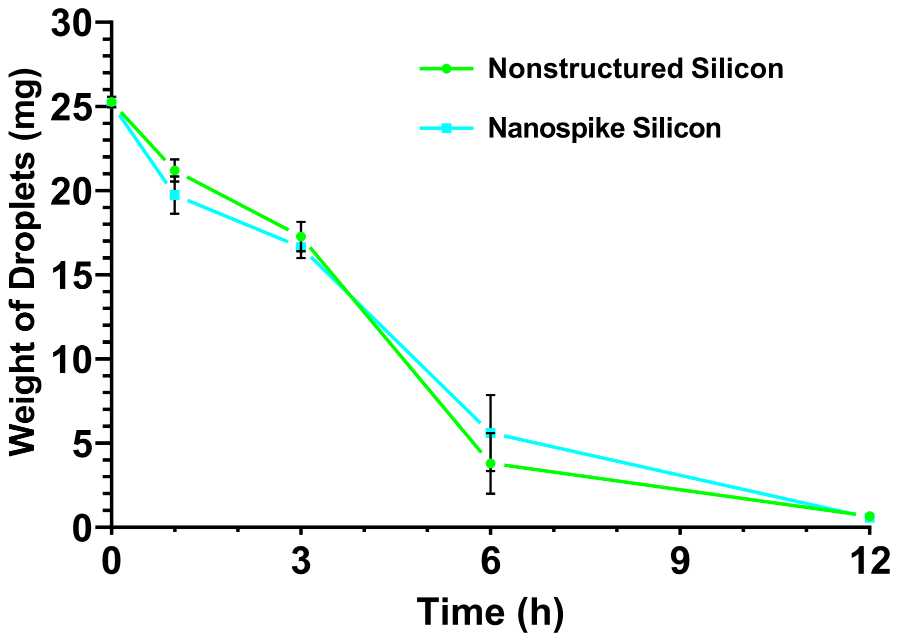

Figure S6. Evaporation kinetics of viral droplets on surfaces. 25 μL of viral maintenance media was dispensed on nonstructured silicon and nanospike silicon surfaces to measure the comparative desiccation rate of the viral droplets on surfaces. The curve represents the weight of the droplet measured at each timepoint. n=3.

Supplementary Methods

RT-qPCR

Table S2. Table of known extracted hPIV-3 RNA copies and their respective $\mathbf{C_t}$ -values. The data are then used to generate a standard curve in Figure S7 below.

**Samples Name**	**C_t value (Triplicates)**			**Average C_t Values**	**Known RNA Copies/ µL**	**Log RNA Copies/µL**
hPIV-3_RNA_10^8	16.000	16.479	16.795	16.425	100000000	8
hPIV-3_RNA_10^6	22.677	22.615	22.821	22.704	1000000	6
hPIV-3_RNA_10^4	29.690	29.781	29.548	29.673	10,000	4
hPIV-3_RNA_10^2	36.529	36.825	36.404	36.586	100	2
hPIV-3_RNA_10^0	38.329	>40*	>40*	38.329	1	0

* Fluorescence reading exceeds 40 cycles indicates that the amplification signal is too weak or undetectable, and the $\mathbf{C_t}$ value cannot be accurately determined. In such cases, the result is considered indeterminable or non-detectable.

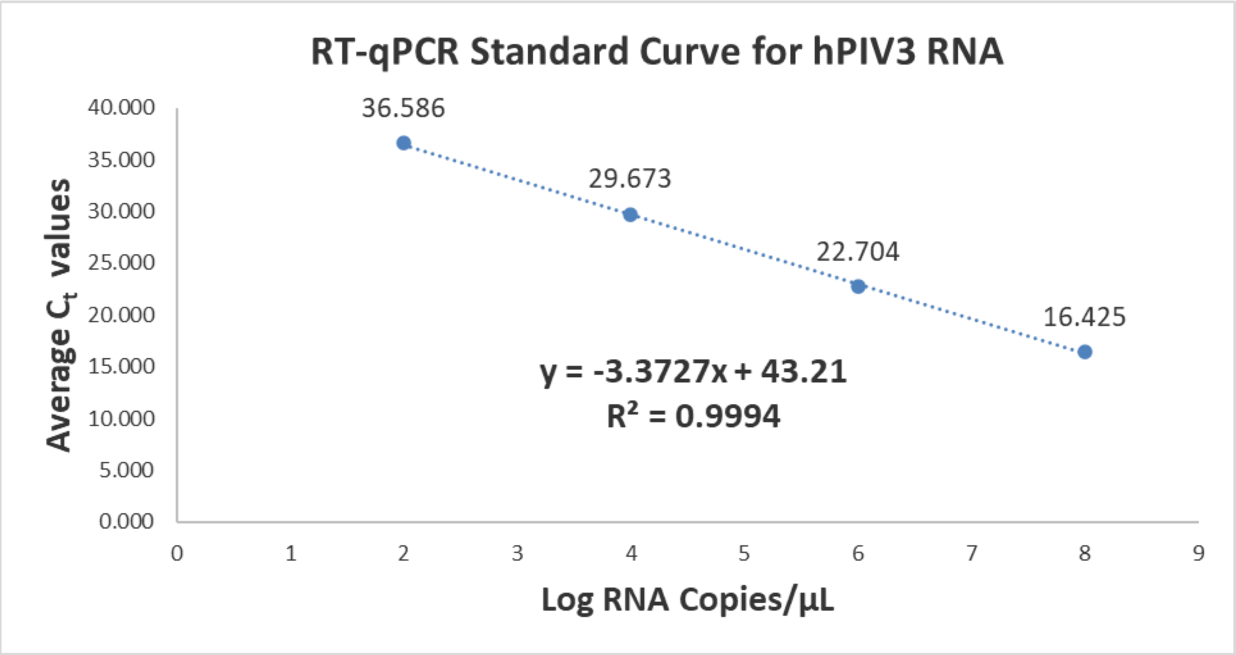

Figure S7. Standard curve generated from extracted hPIV-3 RNA serial dilutions. The equation of the optimum standard curve in (b) is shown as follows: y = -3.3727 x + 43.21, which was constantly used for the extrapolation of viral copies during the antiviral assay. The R square value was closed to 1 and PCR efficiency is 99%, indicating the accuracy of the standard curve generated. Viral copy number = $\mathbf{10}^{\frac{Ct\,T - c)}{k}}$ where Ct T is the threshold crossing value of viral RNA the particular sample; c is the y-intercept, and k is the slope of the linear regression for Ct-value versus the logarithm of the viral RNA copy numbers.

Measurements of the desiccation rate of droplets on surfaces.

Nonstructured Si and Nanospike Si surfaces are measured of their weight on analytical balances 3 times for each replicate. Then, 25 μL of virus maintenance media is dropped onto each individual surface and the total weight of individual surface + viral maintenance media is measured (x3 times). The volume of the viral maintenance media is determined by subtracting the total weight with the weight of the surface and is expressed as milligram (mg).

As desiccation may play a role in inactivating viral particles due to capillary action, we measured the weight of the viral droplets before and after incubation using viral maintenance media (DMEM + 1.6% Trypsin) as mimic. Indeed, volume of viral droplets on surfaces reduces overtime as the weight of the viral maintenance media droplets decreased as time goes by. Interestingly, at 6h, volume of droplets evaporated is higher on Si surface compared to Nanospike Si. It is important to note that the weight of the droplets at 12 h is similar to what was measured at 24 h and 48 h, even though it was visually completely dried.

A droplet (25 uL) of hPIV-3 suspension were incubated on each type of surfaces for 1, 3 and 6 h at ambient environment in dark. The surface area (mm²) of the droplets was calculated using Image J and cross-referenced with formula for area of circle: $A = \pi r^2$. The area of this droplet prints was

then used for estimation of virucidal activity per surface area V; $V = \frac{S - a}{A}$, whereby S is viral titre of stock, a is viral titre of retrieved viral suspension.

References

(1) Wu, S. *Polymer Interface and Adhesion*. 1st Eds. Marcel Dekker: New York, **1982**.

(2) Kim, J. K.; Cho, H. S.; Jung, H.-S.; Lim, K.; Kim, K.-B.; Choi, D.-G.; Jeong, J.-H.; Suh, K.-Y. Effect of Surface Tension and Coefficient of Thermal Expansion in 30 nm Scale Nanoimprinting with Two Flexible Polymer Molds. *Nanotechnology* **2012**, *23* (23), 235303. DOI: 10.1088/0957-4484/23/23/235303.

(3) Hejda, F.; Solar, P.; Kousal, J. Surface Free Energy Determination by Contact Angle Measu Rements -a Comparison of Various Approaches. *WDS'10 Proceedings of Contributed Papers, Part III.* **2010**. 25-30.

(4) Rogachev, A. A.; Tamulevičius, S.; Rogachev, A. V.; Prosycevas, I.; Andrulevičius, M., Features of Polytetrafluoroethylene Coating Growth on Activated Surfaces from Gas Phase. In *Interface Controlled Organic Thin Films*, Al-Shamery, K.; Horowitz, G.; Sitter, H.; Rubahn, H.-G., Eds. : Springer, Berlin, Heidelberg, **2009**; pp 85-89.

(5) Maeda, N.; Israelachvili, J. N.; Kohonen, M. M. Evaporation and Instabilities of Microscopic Capillary Bridges. *Proc. Natl. Acad. Sci. U. S. A.* **2003**, *100* (3), 803-808. DOI: 10.1073/pnas.0234283100.

(6) Klug, W. S.; Bruinsma, R. F.; Michel, J. P.; Knobler, C. M.; Ivanovska, I. L.; Schmidt, C. F.; Wuite, G. J. Failure of Viral Shells. *Phys. Rev. Lett.* **2006**, *97* (22), 228101. DOI: 10.1103/PhysRevLett.97.228101.
